# Resveratrol-activated PI3K/AKT/mTOR signaling pathway attenuates ferroptosis by promoting NRF2/GPX4 metabolic pathway to mitigate IFN-gamma-mediated aplastic anemia

**DOI:** 10.1186/s13020-026-01462-5

**Published:** 2026-07-08

**Authors:** Erhao Zhang, Jianan Cui, Xinran Zhu, Juan Chen, Liqin Xu, Xiaomin Li, Yufei Zhang, Hui Jia, Xiaorong Zhou, Zenghua Lin, Han Wang, Hong Liu

**Affiliations:** 1 Department of Hematology, Affiliated Hospital of Nantong University, Medical School of Nantong University, Nantong, 226001 People’s Republic of China; 2 Department of Immunology, Medical School of Nantong University, Nantong, 226001 People’s Republic of China; 3 Department of Pulmonary and Critical Care Medicine, Affiliated Hospital of Nantong University, Medical School of Nantong University, Nantong, 226001 People’s Republic of China; 4 Department of General Surgery, Affiliated Hospital of Nantong University, Medical School of Nantong University, Nantong, 226001 People’s Republic of China

**Keywords:** Resveratrol, Aplastic anemia, Ferroptosis, NRF2, GPX4

## Abstract

**Background:**

Aplastic anemia (AA) is a severe hematological disorder caused by hyperactivated T cell-mediated hematopoietic failure. It is characterized by hematopoietic stem cell deficiency and hypocellular hematopoiesis in the bone marrow (BM). Ferroptosis, a specific type of programmed cell death, is defined by iron-dependent lipid peroxidation, which has garnered the attention of researchers due to its unique role and biological importance in various diseases. However, the correlation between immunological imbalance-induced ferroptosis and the mortality of hematopoietic stem cells within the BM microenvironment in AA is unclear. Resveratrol (RSV) is crucial in activating NRF2, hence influencing the onset and progression of diseases by regulating ferroptosis. This study aimed to investigate the roles and molecular mechanisms of RSV in the hematopoietic recovery of AA regarding ferroptosis.

**Methods:**

We measured some biomarkers in AA mice and IFN-gamma-treated 32D cells, representing AA syndromes and ferroptosis features. Furthermore, in RSV-treated 32D AA cells, cell activity, cell apoptosis, mitochondrial membrane potential, mitochondrial membrane permeability, and intracellular levels of MDA, ferrous iron, 4-HNE, GSH, ROS, and lipid peroxidation were assessed. Subsequently, additional in vitro and in vivo experiments were performed to investigate the mechanisms by which RSV effectively regulates NRF2 stability, further inhibiting ferroptosis in AA.

**Results:**

We demonstrated that ferroptosis contributes to the occurrence and development of AA disease. RSV dose-dependently inhibits ferroptosis in 32D AA cells by targeting GPX4 expression, enhancing the cell activity of hematopoietic BM cells. Mechanically, RSV significantly increases NRF2 phosphorylation through the PI3K/AKT/mTOR signaling pathway, which maintains NRF2 stability to promote the GPX4 metabolic pathway in AA. Protein levels of p-AKT, p-mTOR, p-NRF2, and GPX4 were markedly increased in BM cells in RSV-treated AA mice, resulting in the suppression of AA development.

**Conclusion:**

Our data demonstrate that RSV effectively inhibits ferroptosis in AA or IFN-gamma-treated 32D cells by targeting the PI3K/AKT/mTOR signaling pathway, which enhances NRF2-mediated GPX4 transcription, thereby ameliorating AA symptoms in vitro and in vivo. This study concludes that RSV is a potential therapeutic drug for ferroptosis-related AA treatment.

**Supplementary Information:**

The online version contains supplementary material available at 10.1186/s13020-026-01462-5.

## Introduction

Aplastic anemia (AA) is a serious immune-mediated bone marrow failure disease, mainly characterized by the pancytopenia in peripheral blood and hypocellular hematopoiesis in bone marrow, and further clinically resulting in purpura, hemorrhage and infrequent infection [1, 2]. A growing body of evidences have shown that AA disease is resulted from a variety of factors and associated with multiple molecular mechanisms [3, 4]. Specifically, the bacterial and viral infections, chemical drugs and radionuclides are known drivers of AA progression, however, the underlying mechanisms driving disease development are not fully known [5]. Over the past decade, growing evidence has revealed that the dysregulation of host immune cells, particularly cytotoxic T cells, dendritic cells, macrophages and nature killer cells, is a crucial culprit for cell-mediated autoimmune attack against hematopoietic progenitor cells [6–9]. Mechanistically, abnormal immune activation in AA patients is attributed to an increased proportion of peripheral blood and bone marrow lymphocytes and an imbalance of T cell subsets, including in particular an increased proportion of helper T cell type 1, CD8^+^ T cells, and γδT cells, while a decrease of regulatory T cells [10]. Meanwhile, a significant increase in the secretion of negative hematopoietic regulators, such as IL-2, IFN-gamma and TNFα, induces the myeloid cell apoptosis, ultimately resulting in the occurrence and development of AA disease [11]. In recent years, there are increasing evidences that the bone marrow hematopoietic microenvironment has an important impact on hematopoietic stem cells, among which IFN-gamma has been considered to be a key cytokine that destroys bone marrow hematopoietic cells, leading to apoptosis of hematopoietic stem cells and bone marrow failure [12, 13].

Currently, hematopoietic stem cell transplantation (HSCT) and immunosuppressive treatment (IST) have been successfully applied to treat AA patients [14, 15]. Although HSCT is widely used to alleviate AA, application of this strategy is clinically restricted attributed from the risk of graft versus host disease and concurrent severe infections after transplantation [16]. Subsequently, IST using immune suppressive medicines, including anti-thymocyte globin and cyclosporin A, has been regarded as the first-line treatment for AA patients via targeting the cytotoxic T cells [17]. Unfortunately, beneficial effects have been obtained in the drug-treated AA, yet two clinical problems are often encountered, namely showing less response and accompanying nephrotoxic and neurotoxic side effects [18]. Therefore, it is necessary to deeply illustrate the molecular mechanisms of AA pathogenesis and explore novel therapeutic strategies for treatment of AA.

Ferroptosis is a novel type of programmed cell death, first reported by Dixon and colleagues in 2012, characterized by iron-dependent accumulation of intracellular reactive oxygen species (ROS) and lipid peroxidation (LIP), playing a vital role in the development of tumors and autoimmune diseases [19, 20]. Interestingly, some physicians have found that AA patients are usually accompanied by significant increases in serum iron ion and ferritin levels, which is not only related to the iron utilization disorders, but also the role of macrophages in iron metabolism. In recent years, numerous investigations have revealed that ferroptosis is an important pathogenesis of hematologic diseases, especially for AA disease. For instance, Liu and coworkers have revealed that panaxadiol saponin (PNDs), a latent targeted drug for the treatment of AA, attenuates ferroptosis in primary megakaryocytes of AA mice complicated with iron-overload and iron dextran-induced Meg-01 cells by activating nuclear factor erythroid 2-related factor 2 (Nrf2)/heme oxygenase-1 (HO-1) and phosphatidylinositol 3-kinase (PI3K)/protein kinase B (AKT)/mammalian target of rapamycin (mTOR) signaling pathways. In addition, their results also demonstrated that PNDs significantly increased percentages of CD4^+^ T cells and CD4^+^/CD8^+^ T cells in the peripheral blood of iron-overloaded AA mice, thereby alleviating the development of AA disease through inhibiting T cells-mediated ferroptosis in bone marrow hematopoietic cells [21]. Additionally, it was corroborated by Zhang et al. that ferroptosis on cytotoxic T lymphocytes (CTLs) may serve as a promising target to against the damage process of bone marrow hematopoietic stem cells in AA patients, thereby exhibiting a more notable effect in alleviating anemia symptoms [22]. Therefore, aplastic anemia, as an autoimmune disease, has a deep understanding of the relationship between aplastic anemia and ferroptosis is crucial for the treatment of this disease.

Resveratrol (3,5,4-trihydroxystilbene, RSV) is a plant polyphenol found in a variety of plants and fruits, such as grapes, peanuts, mulberries, blueberries, pomegranates, and strawberries [23, 24]. Over the past decades, accumulating evidence indicates that RSV has gained attention in ferroptosis regulation for disease therapy due to its multiple biological activities [25]. Recently, as an activator of Nrf2, RSV plays a key role in improving age-related kidney damage by regulating ferroptosis through various pathways, thereby affecting the occurrence and progression of diseases [26]. Interestingly, as a key transcription regulator involved in ferroptotic metabolism, NRF2 restored the intracellular redox homeostasis through the induction of multiple antioxidant response element (ARE)-containing genes, such as glutathione peroxidase 4 (GPX4), a potent enzyme to effectively catalyze glutathione reductase reaction, resulting in the inhibition of ferroptosis [27–29]. For instance, RSV can prevent inflammation and oxidative stress by increasing the activity of NRF2, activating Nrf2/sirtuin 1 (SIRT1) signaling pathway, and alleviating mitigating mitochondrial dysfunction caused by kidney injury and aging [30]. Accordingly, we speculated that the molecular mechanisms by which RSV could effectively treat multiple diseases were closely associated with the ferroptosis signaling pathway. However, RSV for the treatment of aplastic anemia has not been reported, and its target and potential molecular mechanism are still unknown. Therefore, this project will explore the role of RSV on the hematopoietic recovery of AA in the perspective of ferroptosis, and provide a new strategy for the treatment of AA.

In this study, we confirmed that RSV can significantly restore the hematopoietic function of hematopoietic stem cells by inhibiting ferroptosis. Furthermore, RSV can specifically target PI3K protein and promote NRF2 phosphorylation through the PI3K/AKT/mTOR signaling pathway. The phosphorylated NRF2 effectively disrupted the interaction between kelch-like ECH-associated protein 1 (KEAP1) and NRF2, and then elevated the transcriptional level of GPX4, leading to an increase in intracellular GPX4 expression in AA cells. Our data demonstrated RSV treatment could effectively suppress ROS, and malondialdehyde (MDA) and iron ions, yet increase glutathione (GSH) level and mitochondrial membrane potential (MMP), ultimately inhibiting ferroptosis in hematopoietic stem cells. Hence, our findings not only provide an in-depth understanding of the pathogenesis of AA but also offer a novel avenue for the comprehensive treatment of AA.

## Methods and materials

### Cell line and culture

Murine myeloid precursor cell line 32D clone3 cells (YS2220C) were purchased from Yaji Biotech Co., Ltd. (Shanghai, China) and cultured and maintained in RPMI 1640 medium supplemented with 10% fetal bovine serum, 1% penicillin/streptomycin and 10 ng/mL IL-3 (ThermoFisher Scientific, 213-13-50UG) in a humidified atmosphere with 5% CO_2_ at 37 °C.

### Reagents and antibodies

10 mM Resveratrol (RSV) was purchased from MedChemExpress (HY-16561) (MCE, Shanghai, China). RSL3 (HY-100218A), Ferrostatin-1 (Fer-1, HY-100579), Z-VAD-FMK (zVAD, HY-16658B), 3-Methyladenine (3-MA, HY-19312), BAY 11–7082 (BAY, HY-13453), Necrosulfonamide (NSA, HY-100573), H2DCFDA (HY-D0940), BODIPY 581/591 C11 (HY-D1301), JC-1 (HY-15534), Cycloheximide (CHX, HY-12320), MG132 (HY-13259), Chloroquine (CQ, HY-17589A), Bafilomycin A1 (Baf A1, HY-100558), mTOR inhibitor-8 (mTORi, HY-131344), AKT-IN-6 (AKTi, HY-19982), and Pronase (HY-114158) were purchased from MCE (Shanghai, China). RPMI 1640 medium (BC-M-017), FBS (BC-SE-FBS01), Phosphate buffer saline (BC-BPBS-11), and Trypsin–EDTA solution (BC-CE-005) were obtained from Bio-channel (Nanjing, China). The penicillin/streptomycin (C100C5), RIPA buffer (WB3100), IP lysis buffer (P70100), and protease and phosphatase inhibitor (P002) were purchased from NCM (Suzhou, China). RNA isolater Total RNA Extraction Reagent (R401-01), HiScript II Q RT SuperMix for qPCR (R223-01), ChamQ Universal SYBR qPCR Master Mix (Q711-02), CCK-8 Cell Counting Kit (A311-01), protein A/G magnetic beads (PB101-01), Annexin V-PE/7AAD Apoptosis Detection Kit (A213-01), Hyperactive pA/G-MNase CUT&RUN Assay Kit for PCR/qPCR (HD103-01), and dual Luciferase Reporter Assay Kit (DL101-01) were purchased from Vazyme (Nanjing, China). GSH and GSSG Assay Kit (S0053), MDA Assay Kit (S0131S), and LDH Cytotoxicity Assay Kit (C0016) were obtained from Beyotime. 4-HNE(4-Hydroxynonenal) ELISA Kit (E-EL-0128), Ferrous Iron Colorimetric Assay Kit (E-BC-K773-M), IFN-gamma ELISA Kit (E-EL-H0108 and E-EL-M0048), and Glutathione Peroxidase 4 (GPX4) Activity Assay Kit (E-BC-K883-M) were purchased from Elabscience. The antibodies against ACSL4 (22401-1-AP), FSP1 (20886-1-AP), ACSL3 (20710–1-AP), CD98 (15193-1-AP), FPN1 (26601-1-AP), FTH1 (11682-1-AP), SLC7A11 (26864-1-AP), GPX4 (30388-1-AP), PTGS2 (27308-1-AP), KEAP1 (10503-2-AP), Ubiquitin (10201-2-AP), AKT (10176-2-AP), Phospho-AKT (Ser473) (80455-1-RR), mTOR (81670-1-RR), mTOR (81670-1-RR), Phospho-mTOR (Ser2448) (67778-1-Ig), PI3K (20584-1-AP), PTEN (60300-3-Ig), CD34 (83713-2-RR), HA (51064-2-AP), and IgG (30000-0-AP), GAPDH (10494-1-AP) were purchased from Proteintech. The antibodies against CD71 (13113), NRF2 (12721) and NRF2 (40969) were purchased from Cell signaling Technology. The first antibody against KEAP1 (MA5-17106), Alexa Fluor 488 Goat anti-Rabbit IgG second antibody (A-11008), Phospho-Nrf2 (Ser40) Polyclonal Antibody (PA5-67520), and Lipofectamine^®^2000 transfection reagent (11668019) were purchased from ThermoFisher Scientific. The fluorescent antibodies for flow cytometry assays, including FITC-CD3e (553,062), Alexa Fluor 700-CD4 (557956), and BV510-CD8α (563068), were purchased from BD Pharmingen. SABC (Rabbit IgG)-POD kit (SA0021) was purchased from Solarbio.

### Cell viability

For cell viability assays, 3000 cells/well were seeded in a 96-well plate and subjected to various drug treatments, thereafter incubated with 100 μL 1640 medium containing 10% Cell Counting Kit-8 (CCK-8) reagent for 2 h at 37 ℃. The absorbance was measured using a microplate reader at an absorbance of 450 nm (BioTEK Instruments, Inc.).

### Flow cytometry assays

For ROS and LIP assays, primary hematopoietic stem cells, 32D cells, IFN-gamma-treated 32D (32D AA) cells, or GPX4-deficient 32D cells were seeded in 6-well plates and treated with various concentrations of IFN-gamma or RSV for 24 h. All cells were collected and resuspended in serum-free medium containing 2 μM H2DCFDA or 5 μM BODIPY 581/591 C11 to detect the fluorescence levels of ROS or LIP, respectively, using a flow cytometry assay.

For cell apoptosis assays, 32D or 32D AA cells were seeded at 5 × 10^5^ cells per well in 6-well plates and then cultured in RPMI 1640 with various concentrations of IFN-gamma or RSV for 24 h. According to the manufacturer’s protocol, the cells were stained using a PE-Annexin V/7-AAD Apoptosis Detection Kit. Subsequently, apoptotic cells were measured using a flow cytometry assay.

For JC-1assays, 32D AA cells were seeded at 5 × 10^5^ cells per well in 6-well plates, and then cultured in RPMI 1640 with various concentrations of RSV for 24 h. The cells were then stained with JC-1 at 37 °C for 30 min. The fluorescence of JC-1 monomers and aggregates was detected using a flow cytometry assay.

For protein expression assays, 32D AA cells were seeded at 5 × 10^5^ cells per well in 6-well plates, and then cultured in RPMI 1640 with various concentrations of RSV for 24 h. The cells were collected and incubated with first antibody for 12 h at 4 °C. On the second day, these cells were incubated with Alexa Fluor 488 secondary antibody for 1 h at room temperature (RT). The fluorescence of NRF2 or KEAP1 proteins in RSV-treated 32D AA cells was measured using a flow cytometry assay. For the analysis of the proportion of CD8^+^ T and CD4^+^ T cells in peripheral blood, peripheral blood mononuclear cells were collected and stained with CD8 and CD4 antibodies according to the manufacturer’s protocol. Finally, the percentages of T cell subsets were measured using a flow cytometry assay.

### Transmission electron microscopy (TEM)

After IFN-gamma or RSV treatment, 32D cells or 32D AA cells were collected, and then fixed with 2.5% glutaraldehyde for 2 h at 4 ℃. TEM imaging for detecting the mitochondrial morphology was performed by Lilai Biotechnology (Chengdu, China).

### Enzyme-linked immunosorbent assays (ELISA)

Briefly, all cells were collected and then lysed by sonication. The harvested supernatant was used to detect the levels of malondialdehyde (MDA), ferrous iron (Fe^2+^), 4-hydroxynonenal (4-HNE) and glutathione (GSH) using ELISA assays according to the manufacturer’s protocols. The concentration of IFN-gamma in the serum and supernatant of bone marrow flushing fluid was determined by the corresponding ELISA kit. For GPX4 enzyme activity, 32D AA cells treated with RSV were lysed with IP cell lysate to obtain the supernatant. Finally, the enzymatic activity of GPX4 was determined according to the manufacturer’s protocol.

### Western blotting (WB) assays

For cells and mouse tissues, protein samples were harvested using RIPA lysate supplemented with 1% protease and phosphatase inhibitor and denaturized by heating with 5 × loading buffer. The target proteins were separated using SDS-PAGE gels and then transferred to PVDF membranes, followed by blocking with 5% skim milk. Following incubation with the primary antibody and associated second antibodies, protein expression was detected using the ECL reagent, and quantified by the ImageJ software.

For immunoprecipitation (IP) assays, cell lysates were precleared using protein A/G magnetic beads and IgG antibodies, followed by overnight incubation at 4 ℃ with primary antibodies, including NRF2 and KEAP1 antibodies. The next day, 40 μL of protein A/G magnetic beads were introduced and incubated at room temperature for 30 min to ensure complete association of the magnetic beads with the antibody. The magnetic separator facilitated the complete adsorption of magnetic beads, resulting in the enrichment of the protein complexes containing the target molecule. After washing three times with the cell lysis buffer, the supernatants containing protein complexes were obtained by removing the magnetic beads. The target proteins were detected using WB analyses.

### RNA isolation and relative quantitative real-time PCR assays

Total RNA was extracted using a trizol reagent, and reversely transcribed into cDNA according to the manufacturer’s protocol, followed by qPCR analysis for indicated genes using the ABI 7300 Real-time PCR instrument. The fold difference in gene expression was calculated using the comparative Ct (2-^∆∆^Ct) method. All primers were shown in Supplemental Table S1.

### Protein stability and ubiquitination assays

For the protein stability assay of NRF2, 32D AA cells were cultured in 6-well plates. After treatment with 20 μM RSV, the cells were treated with 50 μg/mL cycloheximide (CHX) and subsequently harvested at the indicated time points to extract total protein. Subsequently, with the pre-treatment of 10 μM MG132, 30 nM bafilomycin A1 (Baf A1) or 10 μM chloroquine (CQ), the cells were cotreated with indicated concentrations of RSV for 24 h. The levels of NRF2 protein were evaluated using WB assays.

For ubiquitination assays, 32D AA cells were seeded in 10 cm culture dishes overnight and subsequently treated with indicated concentrations of RSV for 24 h. After IP assays with the NRF2 antibody or IgG antibody, the protein ubiquitination levels were assessed using WB assays.

### Plasmid transfection

For the knockdown of GPX4 or NRF2, siGPX4 for GPX4 (si-GPX4) or siRNA for NRF2 (si-NRF2) were constructed by GenScript, respectively. For the knockout of NRF2, sgRNAs for NRF2 were constructed, and then connected to the lentiCRISPRv2 plasmids (Miaoling Biotechnology). All primers were shown in Supplemental Table S1. In addition, other plasmids used in this research were constructed by Miaoling Biotechnology, including NRF2-FL, NRF2-S40A, HA-NRF2-FL, and HA-NRF2-S40A. Plasmids were transfected into 32D AA cells using Lipofectamine^®^2000 transfection reagent according to the manufacturer’s instructions.

### Proteomics LC-MS/MS and data analysis

Total protein was extracted from 32D AA cells with or without treatment of 20 μM RSV for 24 h. Library construction and LC-MS/MS-based quantitative proteomics were performed by Novegene Company (Beijing, China).

### CUT&RUN assays and luciferase reporter assays

In 32D AA cells, CUT&RUN assays were performed using the Hyperactive pG-MNase CUT&RUN Assay Kit according to the manufacturer’s instructions. CUT&RUN and input DNA were subsequently purified and analyzed using quantitative polymerase chain reaction (qPCR). The value of enrichment was calculated relative to input and the ratio to IgG.

For luciferase reporter assays in 32D cells, the promoter region (−2000 − 10) of GPX4 was amplified by PCR from the mouse genomic DNA and subsequently subcloned into the PGL3-basic vector. Briefly, 3 × 10^4^ 32D cells/well were seeded in 24-well plates and subsequently transfected with the PGL3-GPX4/promoter reporter and renilla plasmids. Following transfection with or without NRF2 plasmids, the cells were subjected to different concentrations of RSV for an additional 24 h. The firefly luciferase and renilla luciferase activities were measured using a dual Luciferase Reporter Assay Kit, following the instructions provided in the protocol. All primers are illustrated in Supplemental Table S1.

### Molecular docking

Molecular docking analysis was performed using AutoDock Vina 1.2.3 to investigate the binding interaction between RSV (PubChem CID: 445154) and phosphatidylinositol 4,5-bisphosphate 3-kinase catalytic subunit alpha isoform (PIK3CA) protein (PDB ID: 9CMV). PyMOL 2.5.5 was used to remove water molecules and ligands from the protein, and the processed file was then saved in PDB format. The Getbox plugin was used to obtain the parameters for the binding pocket. Next, the processed protein file and the active compound file were imported into ADFRsuite 1.0 and converted to PDBQT format. Finally, PyMOL 2.5.5 was used to visualize the results of the molecular docking.

### Cellular thermal shift assay (CETSA)

Briefly, 32D AA cells were incubated with or without RSV for 24 h, then harvested and subjected to PBS supplemented with protease inhibitors. The cell lysates were equally divided into multiple PCR tubes and heated at varying temperatures (37, 41, 45, 49, 53, 57, 61, 65, 69, and 72 ℃) for 3 min, and then immediately placed on ice. Additionally, 32D AA cells were treated with various concentrations of RSV, including 0.01, 0.05, 0.1, 0.5, 1, 5, 10, 20, and 40 µM. Then, the cell lysates in each sample were split into two groups, and then incubated with 41 or 61 ℃ for 3 min, respectively. All cells were lysed, followed by three cycles of freeze-thawing with liquid nitrogen. Finally, proteins were analyzed using WB assays.

### Drug affinity responsive target stability (DARTS)

Cellular extracts from 32D AA cells were incubated in the presence of RSV (0, 10, 20, 30, 40, and 50 μM) for 1 h at room temperature. Then, pronase (0.3 μg/mL) was added to the samples of the indicated groups, and the mixture has been incubated for 15 min at room temperature. The samples were immediately boiled after the addition of loading buffer to stop digestion, and the results were analyzed using WB assays.

### Surface plasmon resonance (SPR)

The binding activity between RSV and PI3K was detected by SPR binding assays using a Biacore-8 K system and a CM5 sensor. Human PI3K protein was used to immobilize the CM5 sensor. Twofold serial dilutions of RSV ranging from 0.3125 to 10 µM were then passed over the surface of the CM5 series S sensor chip at 30 µL/min for 150 s, followed by dissociation in glycine hydrochloride (10 mM) for 300 s to regenerate the chip. Next, the dissociation constant (K_D_) has been determined.

### Patient cohort and tumor xenografts

Written informed consent for the banking of excess biological samples for basic research purposes was obtained from all 12 patients with AA at diagnosis and from 6 healthy donors (HD). Protocols encompassing all study procedures were approved by the institutional review board of the affiliated hospital of Nantong University. Density gradient centrifugation was employed to isolate bone marrow mononuclear cells (BMMNCs) from the iliac crest aspirates of HDs and patients with AA to assess indicators associated with AA and ferroptosis according to the above protocols.

Six-week-old female Balb/c mice and male C57BL/6 mice were procured from the Laboratory Animal Center of Nantong University (Nantong, China). Here, the procedure of murine AA was established based on that of a previous study [31]. Briefly, the first generation of mice produced by crossing Balb/c mice (♀) and C57BL/6 mice (♂) was named CbyB6F1 mice, which were subsequently pre-irradiated with 5.0 Gy X-rays of total body irradiation (TBI) for 4 h and received 5 × 10^6^ C57BL/6 lymphocytes (lymph node cells) from age- and gender- matched donors through intravenous injection. The mice were kept for another 12 days before the follow-up experiments. Furthermore, in our research, the control mice, named the Normal group, were sham-irradiated using lead-brick shielding and injected with equal volumes of saline. In AA mice and normal mice, some biomarkers of AA and ferroptosis were detected.

To assess the therapeutic function of RSV in AA, 18 AA mice were randomly assigned to 3 groups: (1) RSV treatment group, which was injected with 20 mg/kg RSV in a 100 μL volume, (2) RSV and RSL3 cotreatment group, which was injected with 20 mg/kg RSV and 30 mg/kg RSL3 in a 100 μL volume, and (3) Saline group, which was injected with equal volumes of Saline. Meanwhile, we selected six normal mice to set up a Normal group, which received equal volumes of saline. After 30 days of drug administration, all mice were sacrificed. The mouse's weight was measured every 3 days. At the end of the experiment (D30), the organ index and hematological routine were assessed. Additionally, immunohistochemistry assays were performed to evaluate CD34 expression, and hematoxylin and eosin (H&E) staining assays were performed in femoral BM on day 30 according to the manufacturer’s protocol. The levels of MDA, Fe^2+^, 4-HNE, and GSH were detected using ELISA assays. The percentage of CD8^+^ T and CD4^+^ T cells in peripheral blood using flow cytometry assays.

### Statistical analysis

Statistical significance was determined using GraphPad Prism Software 8.0. Data are represented as mean ± SEM of triplicate experiments. Group differences were assessed using a Student’s t-test or one-way ANOVA test. P < 0.05 was considered statistically significant. In the graphed data n.s., *, **, *** denote p value of > 0.05, < 0.05, < 0.01, and < 0.001.

## Results

### Ferroptosis is strongly associated with AA

**Fig. 1:**
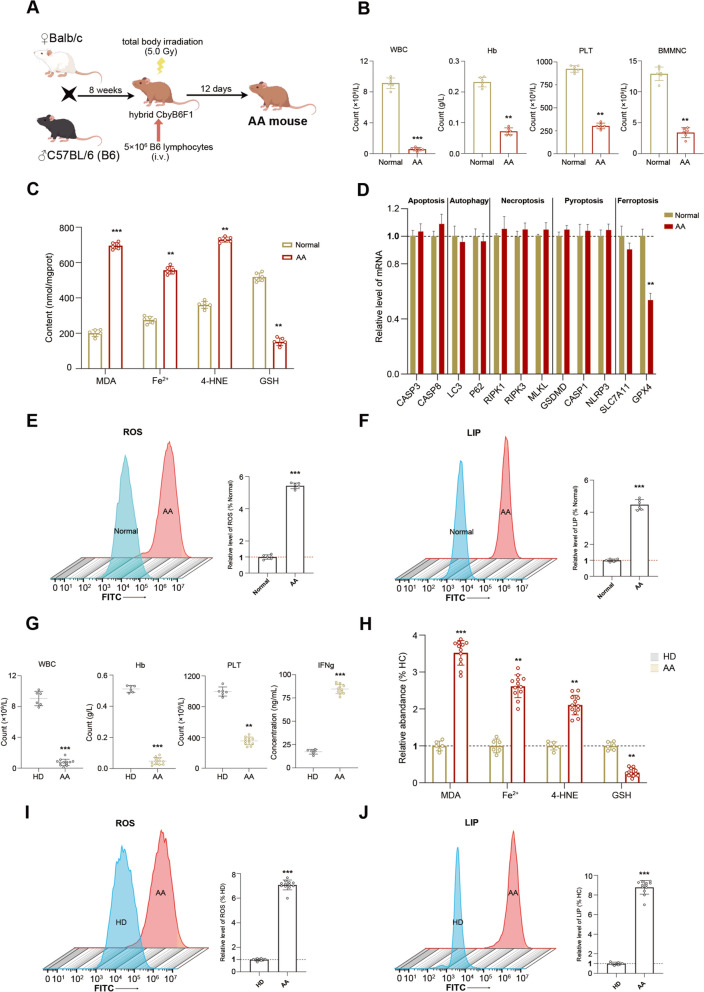
Cytopenias and BM hypocellularity are associated with ferroptosis in AA. **A** Experimental scheme for generating AA mice was used in this research. **B** WBC, Hb, and PLT in peripheral blood and BMMNC derived from BM were measured in AA mice and Normal mice. **C** Ferroptosis-related indicators, such as MDA, Fe^2+^, 4-HNE, and GSH, were detected using ELISA assays in BMMNCs of AA and Normal mice. **D** Levels of gene expression associated with cell death pathways, including apoptosis (CASP3 and CASP8), autophagy (LC3 and p62), necroptosis (RIPK1, RIPK3, and MLKL), pyroptosis (GSDMD, CASP1, and NLRP3) and ferroptosis (SLC7A11 and GPX4) were measured in BMMNCs of AA and Normal mice by qPCR assays. **E**,** F** Flow cytometry assays were performed to detect the level of ROS (**E**) and LIP (**F**) in BMMNCs of AA and Normal mice. **G** WBC, Hb, PLT, and IFN-gamma in peripheral blood were measured in patients with AA and HD. **H** Levels of MDA, Fe^2+^, 4-HNE, and GSH were detected using ELISA assays in BMMNCs of patients with AA and HD. **I**, **J** Flow cytometry assays were performed to detect the level of ROS (**I**) and LIP (**J**) in BMMNCs of patients with AA and HD. Data are presented as mean ± SEM; n = 6 (**B**–**F**; **G**–**J**, HD) or n = 12 (**G**–**J**, AA). Statistical significance was calculated using the “AA group” against the “Normal/HD group”

We established a mouse model of AA to investigate the association between AA and ferroptosis. Figure 1A depicts the comprehensive procedure for developing the AA mouse. Specifically, Balb/c mice (♀) and C57BL/6 mice (♂) were crossed to produce CbyB6F1 mice, which were subsequently pre-irradiated and administered C57BL/6-derived lymphocytes through intravenous injection. Subsequently, several indicators associated with AA were evaluated to confirm the successful establishment and characterization of the AA mouse model. Utilizing a fully automatic hematology analyzer, we observed a significant decrease in white blood cell (WBC), hemoglobin (Hb), and platelet (PLT) by 12 days post-splenocyte transfer compared with normal mice. Furthermore, BMMNCs significantly decreased in AA mice, preliminarily indicating successful establishment of AA mice (Fig. 1B).

To determine the contribution of ferroptosis to the onset of AA disease, several ferroptosis-related indicators, including MDA, Fe^2+^, 4-HNE, and GSH, were detected using ELISA assays. Our findings demonstrate that the levels of MDA, Fe^2+^ and 4-HNE in AA mice have significantly increased compared to those of normal mice, whereas the GSH level has significantly decreased (Fig. 1C). Subsequently, to identify the primary form of cell death in AA, qPCR assays were performed to assess gene expression targeting different cell death pathways, including apoptosis (CASP3 and CASP8), autophagy (LC3 and p62), necroptosis (RIPK1, RIPK3, and MLKL), pyroptosis (GSDMD, CASP1, and NLRP3), and ferroptosis (SLC7A11 and GPX4). Our results reveal that ferroptosis-related genes (SLC7A11 and GPX4) are significantly reduced in AA mice, with no effect observed on other genes (Fig. 1D). To further confirm that the predominant form of AA-related cell death was ferroptosis, flow cytometry assays were conducted to confirm that ROS and LIP accumulation in AA mice utilizing H_2_DCFDA and BODIPY 581/591 C11 fluorescent probes, respectively (Fig. 1E, F).

To further confirm ferroptosis in AA, we examined the features of ferroptosis in clinical samples from patients with AA (n = 12). Consistent with the findings from AA mice, the level of WBC, Hb, and PLT in patients with AA is much lower than that in HD (Fig. 1G). Notably, the pro-inflammatory cytokine IFN-gamma was significantly increased by approximately fourfold compared to HD, indicating that AA may represent a more severe inflammatory disease (Fig. 1G). Additionally, ELISA and flow cytometry assays were performed to reveal several indicators related to ferroptosis within BM, revealing the accumulation of MDA, iron ions, and 4-HNE; the decreased GSH level; and the significant increase in ROS and LIP (Fig. 1H–J). Recent investigations in our research revealed that the ferroptosis of hematopoietic BM cells typically exacerbates AA progression. Collectively, these results indicate that ferroptosis contributes to AA onset and progression.

### Ferroptosis is strongly associated with IFN-gamma-treated 32D cells

**Fig. 2:**
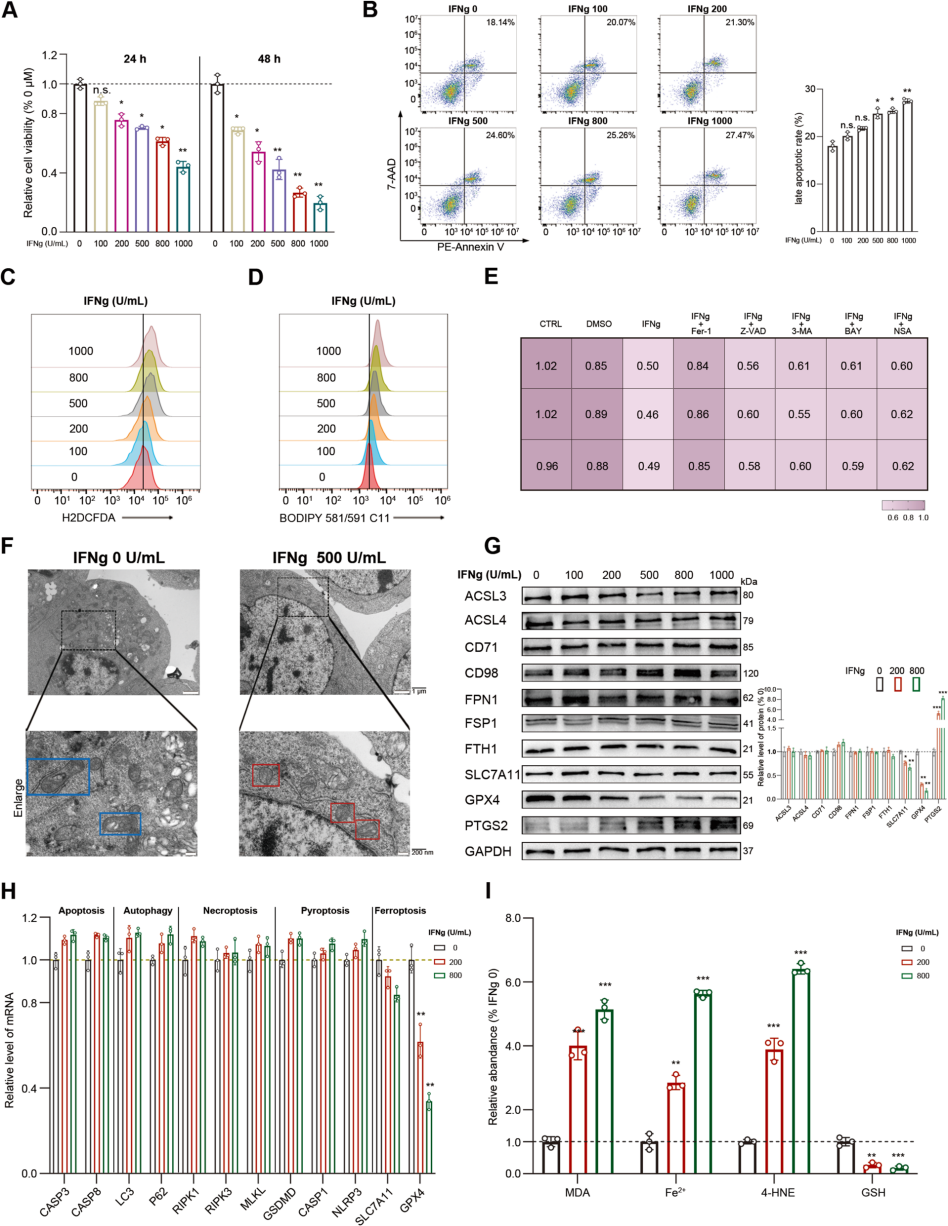
Ferroptosis is associated with decreased cell activity in IFN-gamma-treated 32D cells. **A** CCK-8 assays were performed to detect cell activities of 32D cells in the presence of IFN-gamma. **B** Cell apoptosis in 32D cells in the presence of IFN-gamma was measured by flow cytometry assays. **C**, **D** The levels of ROS (**C**) and LIP (**D**) in IFN-gamma-treated 32D cells were measured by flow cytometry assays. **E** CCK-8 assays were performed to detect cell activities of 32D cells co-treated with IFN-gamma and various inhibitors, including ferroptosis inhibitor (Fer-1), pan-caspase inhibitor (Z-VAD), autophagy inhibitor (3-ME), pyroptosis inhibitor (BAY), and necroptosis inhibitor (NSA). **F** Mitochondrial morphological features in 32D cells treated with IFN-gamma (0 or 500 U/mL) were detected using TEM assays. The blue rectangle indicated intact mitochondria, whereas the red rectangle indicated damaged mitochondria. **G** WB assays were performed to detect the levels of ferroptosis-related proteins in IFN-gamma-treated 32D cells, including ACSL3, ACSL4, CD71, CD98, FPN1, FSP1, FTH1, SLC7A11, GPX4, and PTGS2. **H** qPCR assays were performed to measure the gene expression in IFN-gamma-treated 32D cells, including CASP3, CASP8, LC3, p62, RIPK1, RIPK3, MLKL, GSDMD, CASP1, NLRP3, SLC7A11, and GPX4. **I** ELISA assays were performed to detect the ferroptosis-related indicators in IFN-gamma-treated 32D cells, including MDA, Fe^2+^, 4-HNE, and GSH. Data are presented as mean ± SEM; n = 3. Statistical significance was calculated using the “IFN-gamma-treated group” against the “DMSO group”

To further validate that ferroptosis was the primary cause of cell death in AA, a cell model of AA was established. Previous data indicate that IFN-gamma was significantly increased in AA, suggesting that the IFN-gamma-treated myeloblast-like cell line, 32D, may exhibit AA symptoms. Initially, in 32D cells, several cell activities were assessed following treatment with varying concentrations of IFN-gamma (at doses of 0, 100, 200, 500, 800, and 1000 U/mL), including cell viability and cell apoptosis. CCK-8 assays revealed that IFN-gamma exhibited cytotoxicity to 32D cells in a dose- and time-dependent manner (Fig. 2A). Furthermore, IFN-gamma treatment significantly enhanced cell apoptosis in 32D cells, with 500 U/mL selected for subsequent experiments to establish the AA cell model, termed 32D AA cells (Fig. 2B). To confirm if IFN-gamma may elicit ferroptosis in 32D cells, various ferroptotic events associated with ROS and LIP were detected using flow cytometry. Consequently, compared to the control group (0 U/mL), IFN-gamma treatment significantly increased ROS and LIP levels, corroborating the findings observed in AA mice and patients with AA (Fig. 2C, D; Additional file 1: Fig. S1). For 32D AA cells, a series of rescue experiments was performed using various inhibitors targeting different cell death pathways, identifying the predominant type of cell death in IFN-gamma-induced AA cells. The results demonstrated that only ferrostatin-1 (Fer-1, ferroptosis inhibitor) could mitigate IFN-gamma-induced 32D cell death, whereas Z-VAD-FMK (Z-VAD, pan-caspase inhibitor), 3-methyladenine (3-ME, autophagy inhibitor), BAY 11–7082 (BAY, pyroptosis inhibitor), and necrostatin-1 (NSA, necroptosis inhibitor) had no effect on 32D AA cell activities (Fig. 2E).

Additionally, TEM assays revealed that 32D cells treated with IFN-gamma (500 U/mL) exhibited alterations in mitochondrial morphological features compared with the control cells, including decreased mitochondrial volume, increased bilayer membrane density, and reduced mitochondrial cristae, a unique morphological feature of ferroptosis (Fig. 2F; Additional file 1: Fig. S2). Western blotting analysis revealed that GPX4 level was significantly decreased in a concentration-dependent manner, whereas the expression of cyclooxygenase 2 (COX2 or PTGS2) was increased in 32D cells treated with IFN-gamma (Fig. 2G). These data indicate that the mode of IFN-gamma-induced cell death in 32D cells is dominated by ferroptosis. In addition, qPCR assays revealed that the low expression of SLC7A11 and GPX4 may influence the activity of 32D cells treated with IFN-gamma (Fig. 2H). Lastly, ELISA assays revealed that IFN-gamma significantly increased the levels of MDA, Fe^2+^, and 4-HNE, whereas GSH levels were significantly downregulated in 32D cells (Fig. 2I). Collectively, the above results strongly indicate that ferroptosis is involved in the alteration of the activity of IFN-gamma-treated 32D cells or in AA progression.

### RSV recovers the activity of IFN-gamma-treated 32D cells by inhibiting ferroptosis

**Fig. 3:**
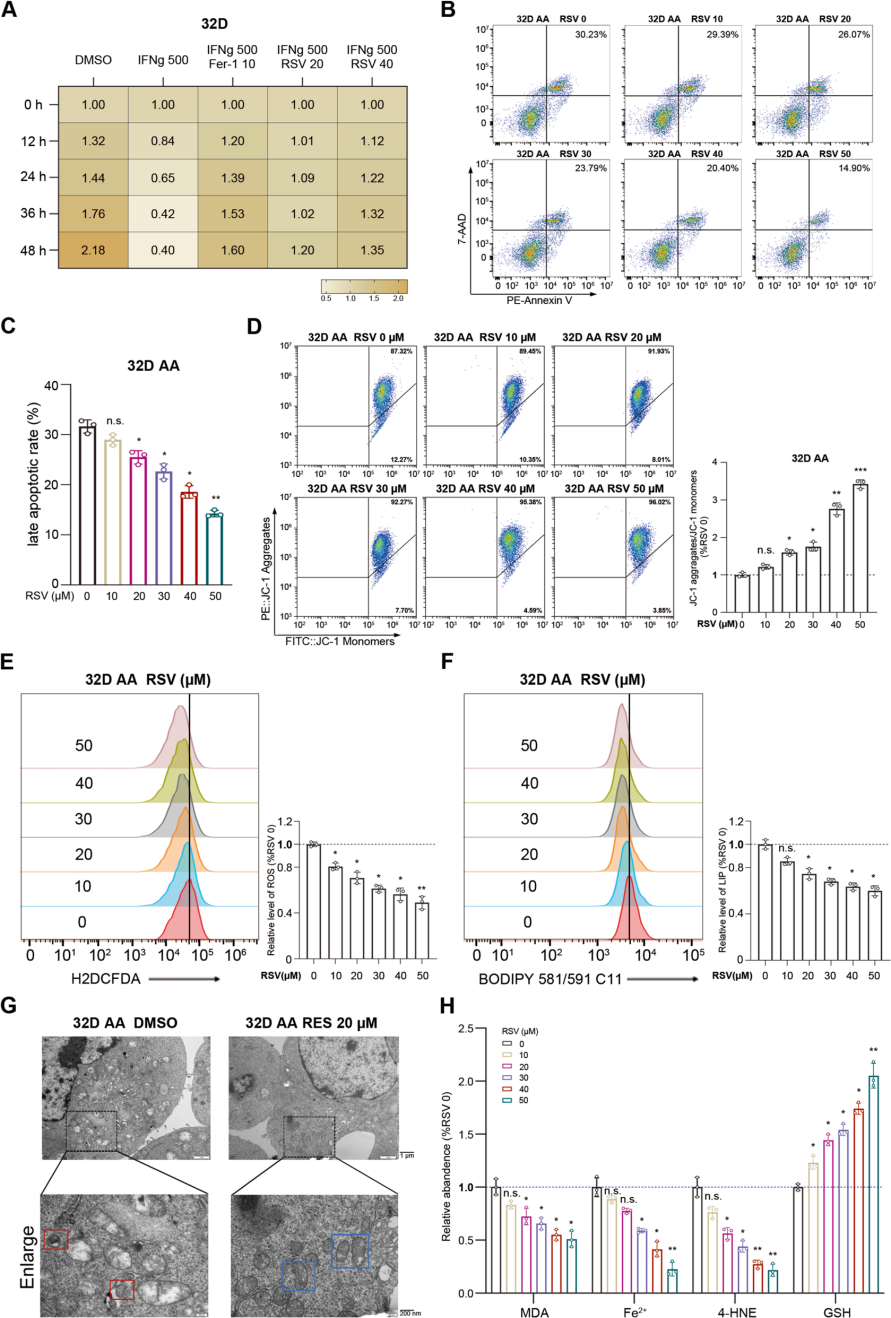
RSV effectively recovers the cell activity by inhibiting ferroptosis in IFN-gamma-treated 32D cells. **A** CCK-8 assays were performed to detect cell activities of 32D cells in the presence of IFN-gamma, Fer-1, and RSV. **B**, **C** Cell apoptosis in 32D AA cells treated with various concentrations of RSV by flow cytometry assays (**B**), and the quantitative data are presented (**C**). **D** Flow cytometry assays were performed to detect MMP in RSV-treated 32D AA cells using JC-1 fluorescent probes, and the quantitative data are presented. **E**, **F** Levels of ROS (**E**) and LIP (**F**) in RSV-treated 32D AA cells were measured using flow cytometry assays, and the quantitative data are presented. **G** TEM assays were performed to detect the mitochondrial morphological features in 32D AA cells treated with RSV (0 or 20 μM). The blue rectangle indicated intact mitochondria, whereas the red rectangle indicated damaged mitochondria. **H** ELISA assays were performed to detect the ferroptosis-related indicators in RSV-treated 32D AA cells, including MDA, Fe^2+^, 4-HNE, and GSH. Data are presented as mean ± SEM; n = 3. Statistical significance was calculated using the “RSV-treated group” against the “DMSO group”

To evaluate if RSV restores the activity of 32D AA cells, we initially investigated the cell viability using CCK-8 assays. Our results revealed that, compared with the control group (DMSO), RSV significantly reversed the inhibition of cell proliferation in 32D AA cells, achieving results similar to those observed in 32D cells treated with IFN-gamma and ferroptosis inhibitor (Fer-1) (Fig. 3A). Furthermore, RSV therapy may somewhat improve cell viability by inhibiting cell apoptosis in 32D AA cells using flow cytometry assays (Fig. 3B, C). Using JC-1 fluorescent probes, we confirmed that RSV treatment induced increased accumulation of JC-1 aggregates (red fluorescence) and lower accumulation of JC-1 monomers (green fluorescence) compared with the control group (DMSO) in 32D AA cells, thereby demonstrating that RSV effectively preserves MMP and permeability (Fig. 3D). To further confirm that RSV inhibits ferroptosis in 32D AA cells, ROS and LIP accumulation were examined using the associated fluorescent probes. The observation indicated that RSV administration effectively suppressed the intracellular accumulation of ROS and LIP (Fig. 3E, F). For the mitochondrial morphology, TEM examination revealed that RSV can mitigate the morphological alterations induced by IFN-gamma treatment, obtaining complete mitochondrial morphology and normal structure (Fig. 3G; Additional file 1: Fig. S3). Additionally, ELISA assays revealed increased levels of MDA, 4-HNE, and iron ions, alongside reduced levels of GSH (Fig. 3H). These findings indicate that RSV dose-dependently inhibits ferroptosis in 32D AA cells, enhancing the cell activity of hematopoietic BM cells and further establishing RSV as a novel agent for the effective treatment of AA.

### RSV inhibits ferroptosis in 32D AA cells through targeting the GPX4 expression

**Fig. 4:**
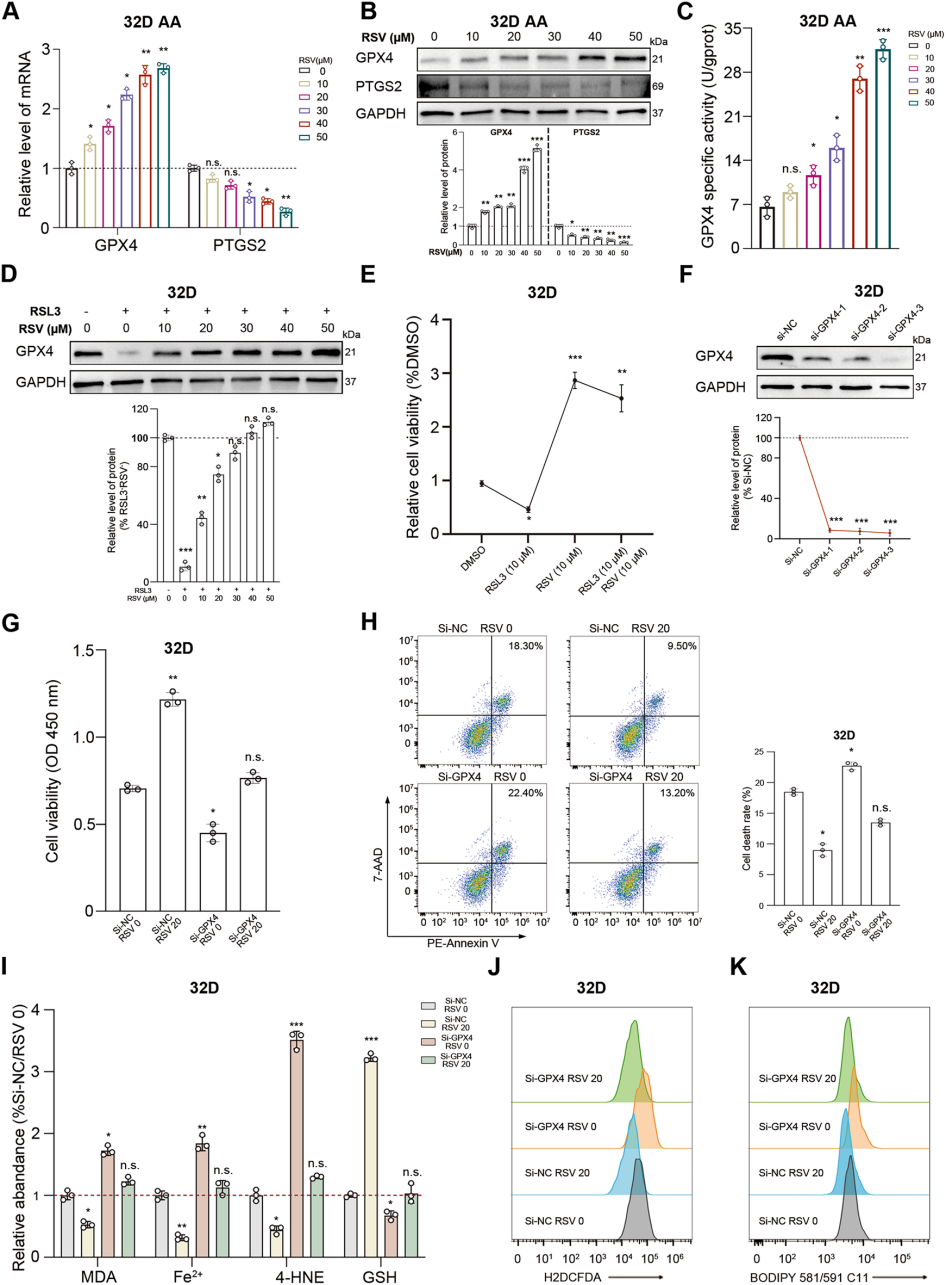
RSV inhibits ferroptosis in 32D AA cells by promoting GPX4 expression. **A** qPCR assays were performed to detect the mRNA levels of GPX4 and PTGS2 in RSV-treated 32D AA cells. **B** WB assays were performed to detect the protein levels of GPX4 and PTGS2 in RSV-treated 32D AA cells. **C** ELISA assays were performed to measure the enzymatic activity of GPX4 in RSV-treated 32D AA cells. **D** WB assays were performed to detect the protein levels of GPX4 in 32D AA cells in the presence of RSV and ferroptosis inducer RSL3. **E** CCK-8 assays were performed to detect cell activities of 32D AA cells in the presence of RSV and RSL3. **F** WB assays were performed in siRNA-NC- or siRNA-GPX4-transfected 32D cells to detect GPX4 expression. **G** CCK-8 assays were performed to detect cell activities of GPX4-deficient 32D cells treated with RSV (0 or 20 μM). **H** Cell apoptosis of GPX4-deficient 32D cells treated with RSV (0 or 20 μM) was assessed by flow cytometry assays, and the quantitative data are presented. **I** ELISA assays were performed to detect the levels of MDA, Fe^2+^, 4-HNE, and GSH in GPX4-deficient 32D cells treated with RSV (0 or 20 μM). **J**, **K** Levels of ROS (**J**) and LIP (**K**) in GPX4-deficient 32D cells treated with RSV (0 or 20 μM) were measured by flow cytometry assays. Data are presented as mean ± SEM; n = 3. Statistical significance was calculated using the “treated/si-GPX4 group” against the “DMSO/si-NC group”

To evaluate if RSV treatment inhibits the cellular ferroptosis of AA, qPCR and WB assays were performed in 32D AA cells treated with various concentrations of RSV, aiming to elucidate the effects of RSV on GPX4 and PTGS2 expression, thereby clarifying the underlying molecular mechanisms by which RSV regulates ferroptosis. The findings revealed that RSV significantly increased the mRNA and protein expression of GPX4, with a significant reduction in PTGS2 expression (Fig. 4A, B). Previous research has revealed that GPX4, a phospholipid hydroperoxide glutathione peroxidase, efficiently inhibits the onset of ferroptosis. Notably, the enzymatic activity of GPX4 in RSV-treated 32D AA cells significantly increased in a dose-dependent manner, further demonstrating that RSV effectively protected GPX4 protein from IFN-gamma-dependent degradation (Fig. 4C). Besides, WB data revealed that the protein accumulation of GPX4 in 32D cells cotreated with RSV and RSL3 was significantly enhanced compared to RSL3 treatment alone, exhibiting notable dose-dependent rescue (Fig. 4D). RSL3 markedly inhibited the activity of 32D cells, whereas RSV significantly reversed this effect (Fig. 4E).

To further confirm that RSV-inhibited ferroptosis relies on GPX4, WB assays revealed that GPX4 knockdown was successfully established in 32D cells (Fig. 4F). CCK-8 assays revealed that GPX4 deficiency inhibited cell activity in 32D cells, which was reversed by RSV treatment, rendering cell activity equivalent to that of the wild type (Fig. 4G). Additionally, flow cytometry assays revealed an increased apoptotic rate in GPX4-deleted 32D cells compared to the wild-type cells, although a significant reduction was observed following RSV treatment (Fig. 4H). The consequences of MDA, Fe^2+^ and 4-HNE accumulation and GSH level reduction in GPX4-deficient 32D cells were ameliorated by RSV treatment (Fig. 4I). Flow cytometry assays using H2DCFDA and BODIPY 581/591 C11 fluorescent probes demonstrated the intracellular accumulations of ROS and LIP in GPX4-deficient 32D cells respectively, whereas RSV treatment restored these levels to normal (Fig. 4J, K; Additional file 1: Fig. S4). Collectively, these results highlight that RSV exerts an inhibited-ferroptotic effect by regulating the GPX4 expression in 32D AA cells.

### RSV-improved the cell activity of 32D AA cells through NRF2/GPX4 signaling pathway

**Fig. 5:**
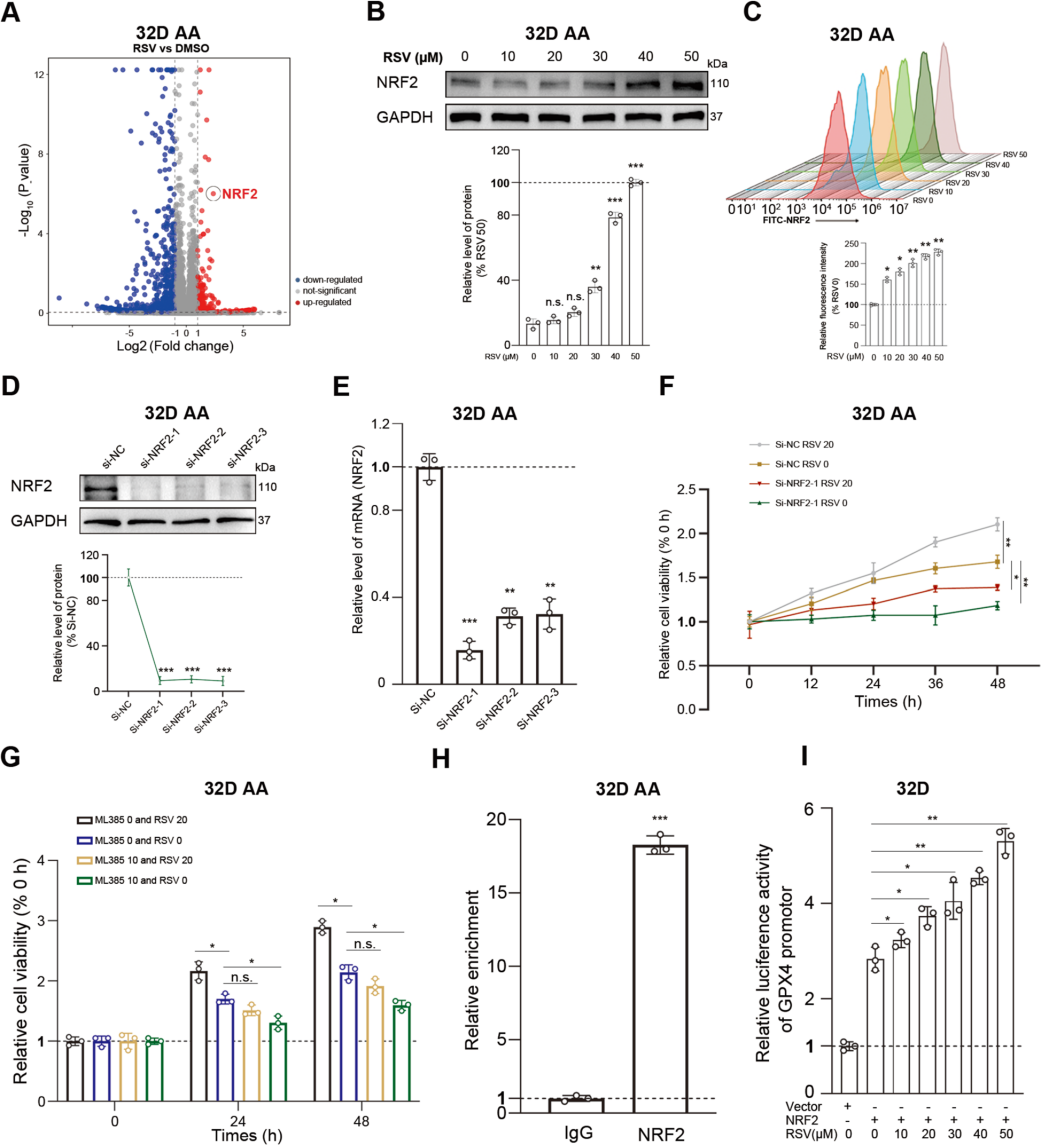
RSV facilitates NRF2/GPX4 upregulation to improve the cell activity of 32D AA cells. **A** Volcano plots displaying differentially expressed proteins between the control group (DMSO) and the treatment group (20 μM RSV) were documented in 32D AA cells. **B** WB assays were performed to detect the protein levels of NRF2 in RSV-treated 32D AA cells, and the quantitative data are presented. **C** Flow cytometry assays were performed to detect the protein levels of NRF2 in RSV-treated 32D AA cells, and the quantitative data are presented. **D** WB assays were performed in siRNA-NC- or siRNA-NRF2-transfected 32D AA cells to detect NRF2 expression, and the quantitative data are presented. **E** qPCR assays were performed to detect NRF2 expression in NRF2-deficient 32D AA cells. **F** CCK-8 assays were performed to detect cell activities of NRF2-deficient 32D AA cells treated with RSV (0 or 20 μM). **G** CCK-8 assays were performed to detect cell activities of 32D AA cells co-treated with RSV (0 or 20 μM) and NRF2 inhibitor ML385 (10 μM) at the indicated times. **H** CUT&RUN assays were performed to explore whether NRF2 regulates the transcriptional level of GPX4 in 32D AA cells. **I** Dual luciferase assays were performed to explore whether RSV has a significant influence on GPX4 expression regulated by NRF2 in 32D cells. Data are presented as mean ± SEM; n = 3. Statistical significance was calculated using the “treated/si-NRF2 group” against the “DMSO/si-NC group”

According to previous research, the transcription factor NRF2, a crucial antioxidant component, plays a significant role in enhancing the transcription of GPX4 to inhibit ferroptosis. As previously confirmed, we initially performed differential protein expression analysis to investigate the mechanism underlying RSV-inhibited ferroptosis in 32D AA cells. In our study, the differentially expressed protein analysis revealed that NRF2 was significantly upregulated in RSV-treated 32D AA cells compared to the control group (DMSO) (Fig. 5A). Herein, we further investigated the effect of RSV on GPX4 expression and the potential molecular mechanism by which RSV facilitates ferroptosis by enhancing GPX4 transcription. Subsequently, in 32D AA cells, WB analysis revealed a significant dose-dependent increase in NRF2 protein following RSV administration (Fig. 5B). Moreover, using FITC-conjugated NRF2 antibodies (FITC-NRF2), flow cytometry assays revealed the enhanced expression of NRF2 protein in 32D AA cells treated with several concentrations of RSV (Fig. 5C). Our results initially revealed that NRF2 may be a potential target for RSV in AA treatment.

To further confirm the potential mechanism by which RSV could effectively decrease GPX4 expression in an NRF2-dependent manner, NRF2 knockdown was established in 32D AA cells (Fig. 5D, E). Subsequently, in NFR2 genetic silencing cells, the cell viability was significantly diminished and effectively reversed following treatment with RSV in 32D AA cells (Fig. 5F). Similarly, the NRF2 inhibitor, ML385, significantly induced 32D cell death. However, RSV treatment could compromise the efficacy of ML385 inhibition, leading to enhanced cell viability and effective treatment of AA caused by NRF2 damage (Fig. 5G). Additionally, CUT&RUN assays reveal an enrichment of GPX4 DNA fragments by anti-NRF2 antibodies in 32D AA cells (Fig. 5H). RSV significantly enhanced GPX4 promoter reporter activity in a dose-dependent manner in 32D cells, as demonstrated by dual-luciferase assays (Fig. 5I). Collectively, our findings indicate that RSV effectively regulates the NRF2/GPX4 axis, contributing to ferroptosis regulation in AA.

### RSV impairs the ubiquitination of NRF2 protein by inhibiting the interaction between KEAP1 and NRF2

**Fig. 6:**
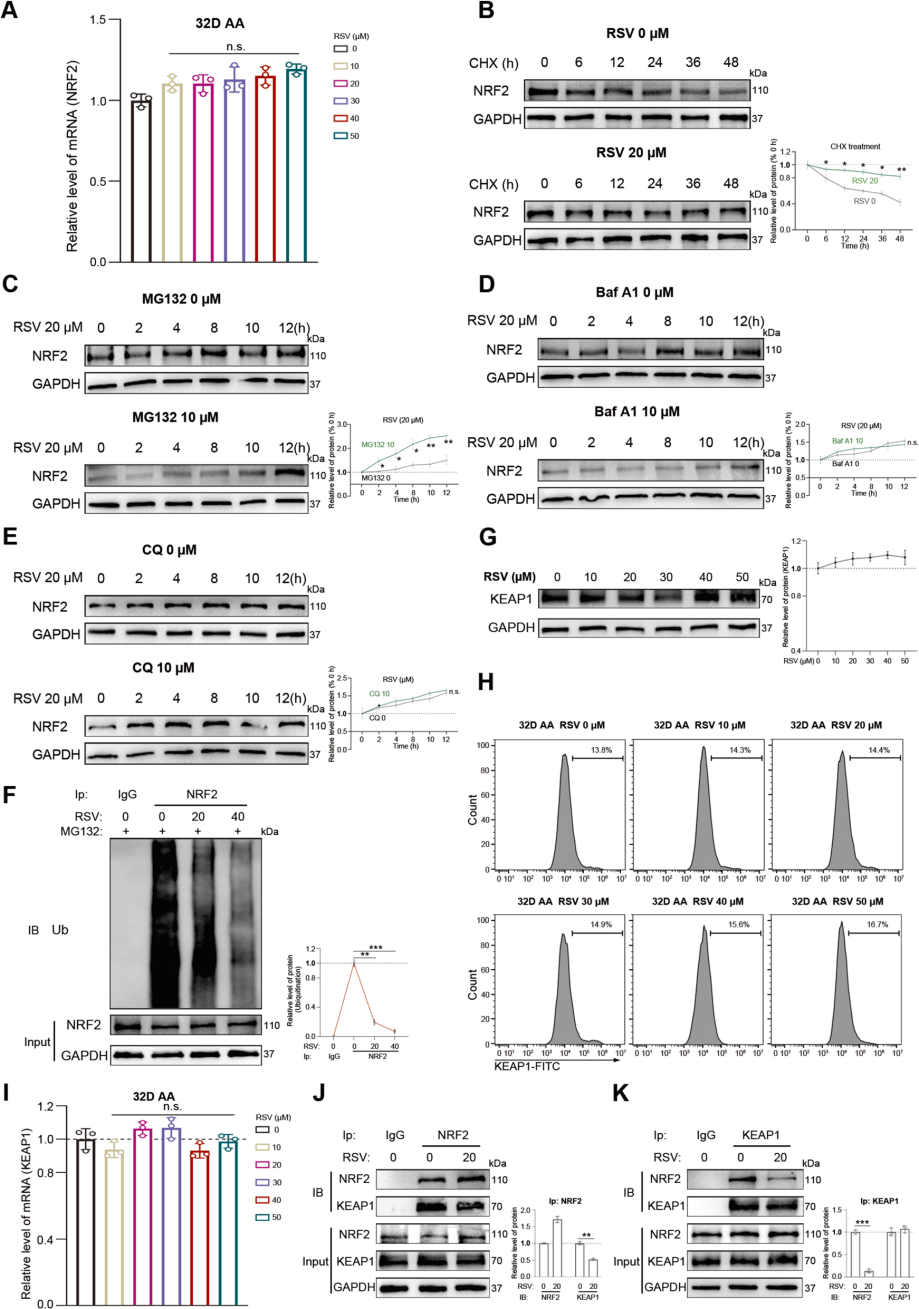
RSV promotes the NRF2 expression by impairing KEAP1-mediated ubiquitination in 32D AA cells. **A** qPCR assays were performed to detect the mRNA levels of NRF2 in RSV-treated 32D AA cells. **B** WB assays were performed to detect the protein level of NRF2 in 32D AA cells treated with or without 20 μM RSV in the presence of CHX (50 μg/mL) for the indicated time intervals, and the quantitative data are presented. **C**–**E** WB assays were performed to detect the protein level of NRF2 in 32D AA cells treated with 20 μM RSV in the absence or presence of MG132 (10 μM) (**C**), Baf A1 (10 μM) (**D**), or CQ (10 μM) (**E**) for the indicated time intervals, and the quantitative data are presented. **F** WB assays were performed to detect the ubiquitination of NRF2 in 32D AA cells in the presence of RSV (20 or 40 μM), and the quantitative data are presented. **G** WB assays were performed to detect the protein levels of KEAP1 in RSV-treated 32D AA cells, and the quantitative data are presented. **H** Flow cytometry assays using FITC-KEAP1 antibodies were performed to detect the protein levels of KEAP1 in RSV-treated 32D AA cells. **I** qPCR assays were performed to detect the mRNA levels of KEAP1 in RSV-treated 32D AA cells. **J**, **K** Immunoprecipitation assays were performed to detect the levels of indicated proteins in 32D AA cells in the presence of RSV (0 or 20 μM) using NRF2 (**J**) or KEAP1 (**K**) antibodies, and the quantitative data are presented. Data are presented as mean ± SEM; n = 3. Statistical significance was calculated using the “RSV-treated group” against the “DMSO group” (**A**, **B**, **F**, **G**, **I**–**K**) and using the “MG132-/Baf A1-/CQ-treated group” against the “MG132-/Baf A1-/CQ-untreated group” (**C**, **D**, **E**)

To clarify the molecular mechanism by which RSV affects NRF2 expression, we further decided to investigate the expression of NRF2 at the mRNA level in 32D AA cells subjected to different doses of RSV. Our results demonstrated that RSV had no significant effect on the mRNA level of NRF2 in 32D AA cells, indicating that RSV may regulate the stability of the NRF2 protein but not its gene transcription (Fig. 6A). After treatment with CHX, a protein synthesis inhibitor, for varying durations NRF2 protein exhibited a shorter half-life in 32D AA cells, indicating that NRF2 protein is unstable and readily degradable in AA disease. These results demonstrated that ferroptosis, resulting from NFR2 deficiency, facilitates the onset and development of AA disease. Notably, in 32D AA cells treated with 20 µM RSV, the protein level of NRF2 exhibited no significant variation across multiple treatment durations, indicating that RSV administration markedly enhanced the stability of NRF2 protein (Fig. 6B). Subsequently, to investigate the potential mechanism by which RSV enhances NRF2 protein stability in 32D AA cells, we utilized MG132, a 26S proteasome inhibitor associated with the proteasome system, or autophagy inhibitors, such as Baf A1 or CQ, to assess the degradation rates of the pre-existing NRF2 protein in RSV-treated 32D AA cells. WB assays revealed that NRF2 protein levels produced by RSV may be significantly increased by MG132 administration compared to the control group (MG132 0 µM) (Fig. 6C). However, no similar results were found in 32D AA cells cotreated with autophagy inhibitors and RSV, indicating no significant correlation between the autophagy degradation pathway and NRF2 protein stability (Fig. 6D, E). Co-immunoprecipitation assays utilizing an anti-NRF2 antibody were performed to assess the ubiquitination status of NRF2, demonstrating that RSV is required for the removal of the polyubiquitinated chain from NRF2 in 32D AA cells and exerts its deubiquitin function (Fig. 6F). Collectively, the above experimental results confirmed that RSV can diminish the ubiquitination of NRF2, enhance protein stability, and inhibit the occurrence of ferroptosis in 32D AA cells.

Previous studies indicate that KEAP1 functions as a key ubiquitinase participating in ferroptosis by facilitating the ubiquitination of NRF2. We further investigated the impact of RSV on KEAP1 expression in 32D AA cells. Our results revealed that RSV has no effect on KEAP1 expression in terms of mRNA and protein (Fig. 6G–I). Therefore, we further hypothesize that RSV facilitates the dissociation of NRF2 and KEAP1 by competitively binding KEAP1. As expected, an immunoprecipitation assay utilizing anti-KEAP1 antibodies or anti-NRF2 antibodies revealed that KEAP1 interacted with NRF2 in 32D AA cells, and RSV significantly impaired their interaction (Fig. 6J, K). Overall, these findings indicated that RSV significantly enhances KEAP1/NRF2 dissociation compared with control (RSV 0 µM) and NRF2 accumulation in 32D AA cells, subsequently improving GPX4 expression to inhibit ferroptosis.

### RSV-increased NRF2 phosphorylation significantly modulates the stability of NRF2 via the PI3K/AKT/mTOR pathway to regulate ferroptosis

**Fig. 7:**
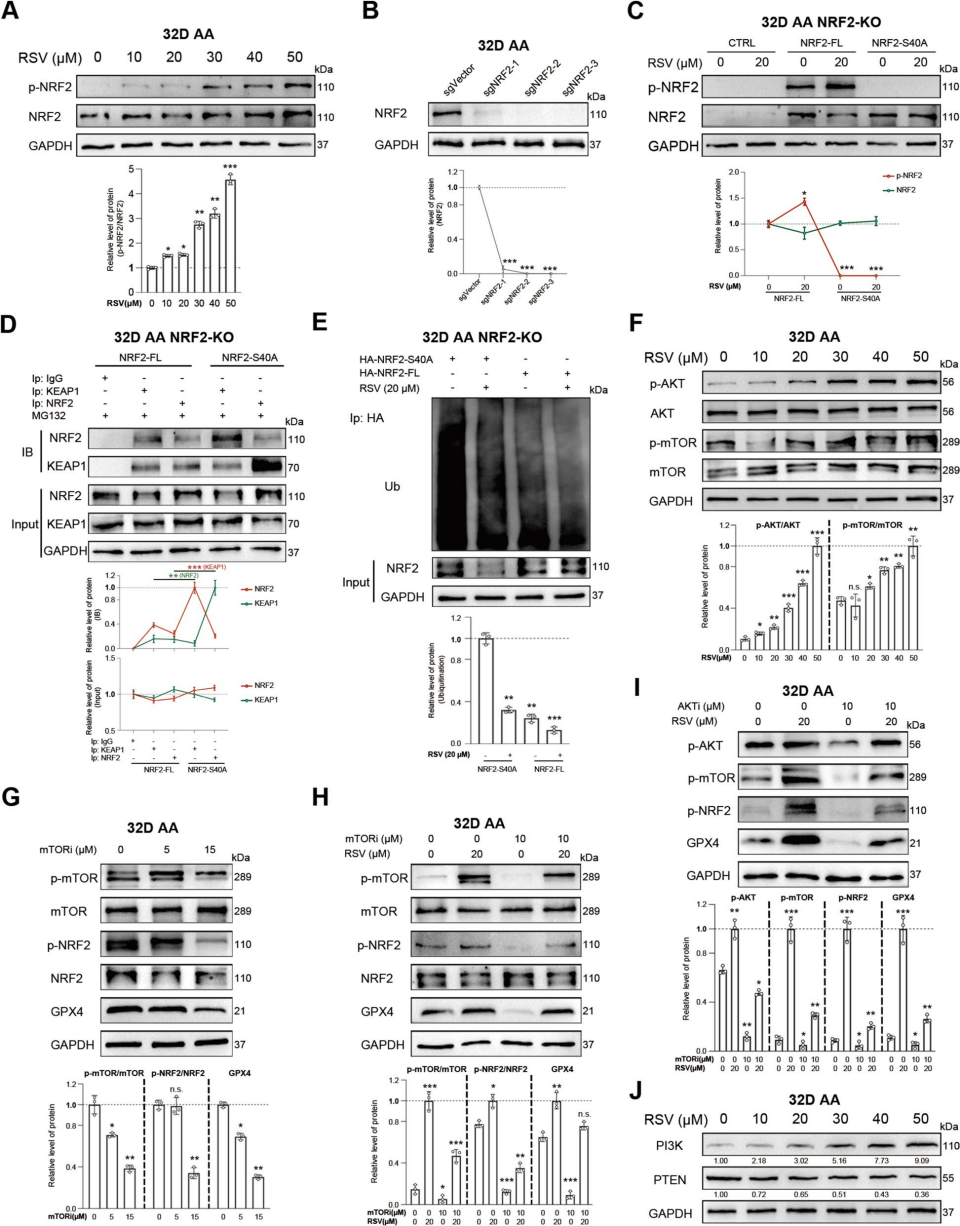
RSV increases the phosphorylation of NRF2 via the PI3K/AKT/mTOR signaling pathway to improve the stability of NRF2. **A** WB assays were performed to detect the protein levels of p-NRF2 and NRF2 in RSV-treated 32D AA cells, and the quantitative data are presented. **B** WB assays were performed in sgVector- or sgNRF2-transfected 32D AA cells to detect NRF2 expression, and the quantitative data are presented. **C** WB assays were performed in full-length NRF2 (NRF2-FL)- or NRF2 mutant (NRF2-S40A)-overexpressing NRF2-deficient 32D AA cells with or without 20 μM RSV treatment to detect the protein levels of p-NRF2 and NRF2, and the quantitative data are presented. **D** Immunoprecipitation assays were performed to detect the levels of indicated proteins in NRF2-FL- or NRF2-S40A-overexpressing NRF2-deficient 32D AA cells in the presence of MG132 (10 μM) using NRF2 or KEAP1 antibodies, and the quantitative data are presented. **E** WB assays were performed to detect the ubiquitination of NRF2 in NRF2-FL- or NRF2-S40A-overexpressing NRF2-deficient 32D AA cells in the presence of RSV (0 or 20 μM), and the quantitative data are presented. **F** WB assays were performed to detect the protein levels of p-AKT, AKT, p-mTOR, and mTOR in RSV-treated 32D AA cells, and the quantitative data are presented. **G** WB assays were performed to detect the protein levels of p-mTOR, mTOR, p-NRF2, NRF2, and GPX4 in 32D AA cells treated with mTOR inhibitors (0, 5, and 15 μM), and the quantitative data are presented. **H** WB assays were performed to detect the protein levels of p-mTOR, mTOR, p-NRF2, NRF2, and GPX4 in 32D AA cells co-treated with mTOR inhibitors (0 and 10 μM) and RSV (0 and 20 μM), and the quantitative data are presented. **I** WB assays were performed to detect the protein levels of p-AKT, p-mTOR, p-NRF2, and GPX4 in 32D AA cells co-treated with AKT inhibitors (0 and 10 μM) and RSV (0 and 20 μM), and the quantitative data are presented. **J** WB assays were performed to detect the protein levels of PI3K and PTEN in RSV-treated 32D AA cells, and the quantitative data are presented. Data are presented as mean ± SEM; n = 3. Statistical significance was calculated using the “treated group” against the “DMSO group” (**A**, **F**–**J**), using the “sgNRF2 group” against the “sgVector group” (**B**), using the “experimental group” against the “NRF2-FL/DMSO group” (**C**), using the “NRF2-S40A group” against the “NRF2-FL group” (**D**), using the “experimental group” against the “NRF2-S40A/DMSO group” (**E**)

To further clarify the molecular mechanism of action of RSV for inhibition of the KEAP1/NRF2 complex, NRF2 phosphorylation was measured using WB assays in 32D AA cells treated with various RSV concentrations. Further immunoblot assay revealed that RSV increased the phosphorylation to total protein ratio of NRF2 in 32D AA cells in a dose-dependent manner, suggesting that RSV-mediated phosphorylated NRF2 plays an important role in alleviating AA (Fig. 7A). Additionally, we have effectively developed NRF2 knockout in 32D AA cells transfected with sgNRF2 plasmids compared with the sgVector-expressing cells (Fig. 7B). To confirm if RSV significantly phosphorylates the Ser40 of NRF2 to activate NRF2, we transfected the NRF2 wild-type (NRF2-FL) and Ser40 mutant (NRF2-S40A, where Ser was mutated as Ala) plasmids into NRF2-deficient 32D AA cells. RSV treatment led to significantly activated NRF2 (p-NRF2) in NRF2-FL-overexpressed 32D AA cells, which was inhibited by NRF2-S40A overexpression (Fig. 7C). Further co-immunoprecipitation experiments using anti-KEAP1 antibodies or anti-NRF2 antibodies indicated that, compared with the NRF2-FL group, the effect of NRF2-S40A mutant on promoting the formation of KEAP1/NRF2 complex was significantly enhanced (Fig. 7D). Subsequently, co-immunoprecipitation experiments using anti-HA antibodies were performed to investigate the effect of NRF2 mutant on the ubiquitination level. Our findings revealed that the interaction between KEAP1 and NRF2 triggered by an NRF2 mutation resulted in a substantial increase in NRF2 ubiquitination, where RSV treatment effectively abolished the effect of NRF2 mutant (Fig. 7E).

The immunoblot assay was performed to examine the activated status of AKT and mTOR to investigate how RSV induces NRF2 phosphorylation at the Ser40 site. Phosphorylation of AKT (p-AKT) and mTOR (p-mTOR) was significantly increased in 32D AA cells treated with various RSV concentrations (Fig. 7F). Subsequently, WB experiments demonstrated that inhibition of the active state of mTOR utilizing the mTOR inhibitor-3 significantly decreased GPX4 protein levels and phosphorylated NRF2 (Fig. 7G), a condition that was ameliorated by RSV treatment (Fig. 7H). Similarly, the administration of AKT inhibitor in 32D AA cells resulted in significantly reduced protein levels of p-AKT, p-mTOR, p-NRF2, and GPX4 compared with the control group, however, the decreasing effects were mitigated by RSV treatment (Fig. 7I). Previous studies have demonstrated that PI3K, a crucial protein phosphatase, can directly phosphorylate Akt1 at S473 and T308, hence enhancing Akt activity through phosphorylation in a PI3K-dependent manner. Therefore, we confirmed that the protein level of PI3K significantly increased by RSV treatment (Fig. 7J). Collectively, these results indicate that RSV significantly increased NRF2 phosphorylation at the Ser40 site through the activation of PI3K/AKT/mTOR/NRF2/GPX4 signaling pathway, inhibiting ferroptosis in 32D AA cells.

### RSV directly interacts with PI3K protein

**Fig. 8:**
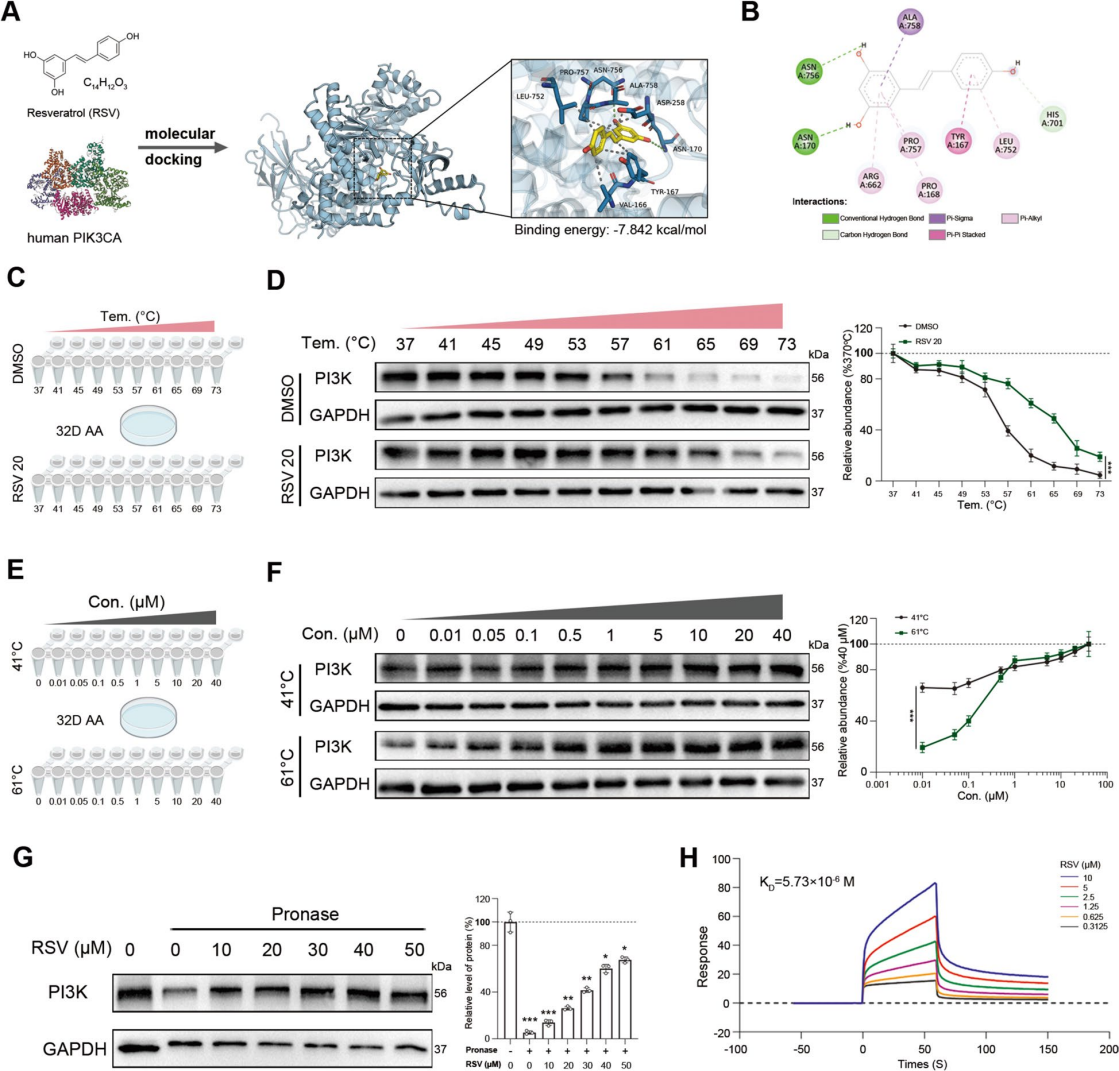
RSV directly interacts with PI3K. **A** 3D binding mode diagram of the compound and protein, where the yellow sticks represent RSV, the blue cartoon represents the phosphatidylinositol 4,5-bisphosphate 3-kinase catalytic subunit alpha isoform (PIK3CA) protein, the green dashed lines indicate hydrogen bonds, and the gray dashed lines indicate hydrophobic interactions. **B** 2D binding pattern diagram of RSV and PIK3CA proteins. **C**, **D** WB assays were performed to evaluate the RSV-promoted thermal stability of PI3K in 32D AA cell lysates at the increasing temperatures up to 73 ℃ were analyzed, and the quantitative data are presented. **E**, **F** WB assays were performed to evaluate the RSV-promoted thermal stability of PI3K in 32D AA cell lysates mixed with the different concentrations of RSV at 41 ℃ and 61 ℃, and the quantitative data are presented. **G** DARTS assays were performed to explore the interaction between RSV and PI3K, and the quantitative data are presented. **H** SPR analyses were performed to analysis of the binding affinity of RSV for PI3K protein. Data are presented as mean ± SEM; n = 3. Statistical significance was calculated using the “treated group” against the “DMSO group” (**D**, **G**) and using the “61 ℃-treated group” against the “41 ℃-treated group” (**F**)

Next, to better explore the interaction modes between RSV (PubChem CID 445154) and PIK3CA protein (PDB ID: 9CMV), we performed molecular docking analysis. The results revealed that there is a robust interaction between RSV and the PIK3CA protein in the presence of stable hydrogen bonds and hydrophobic interactions, with a binding energy of − 7.842 kcal/mol (Fig. 8A). Meanwhile, our data showed that RSV forms a relatively stable binding conformation with the PIK3CA receptor, with the binding pocket mainly involving residues such as VAL-166, TYR-167, ASP-258, LEU-752, PRO-757, and ALA-758 (Fig. 8A). In terms of hydrophobic interactions, the ligand forms extensive hydrophobic interactions with VAL-166, TYR-167, ASP-258, PRO-757, ALA-758, and LEU-752, which improve the fit of the ligand in the binding cavity and reduce its conformational flexibility. At the same time, RSV forms hydrogen bonds with ASN-170 and ASN-756, further stabilizing its orientation within the binding pocket (Fig. 8B). Overall, hydrophobic interactions and hydrogen bonds work together to allow RSV to anchor well to the PIK3CA active site, suggesting that this complex has a relatively stable binding pattern. CETSA was performed on 32D AA cell lysates, and the results demonstrated RSV-induced thermal stabilization of PI3K, with significantly enhanced protein stability over a temperature gradient (49–65 ℃) (Fig. 8C, D). Furthermore, to investigate whether PI3K stability was dose-dependent, dose–response experiments of RSV at 61 ℃ also validated target engagement, showing concentration-dependent PI3K stabilization (Fig. 8E, F). Subsequently, consistent with the thermal stabilization observed in CETSA, we employed DARTS assays, demonstrating that RSV partially blocked protease-mediated degradation of PI3K in a dose-dependent manner (Fig. 8G). In addition, to identify the interaction between RSV and PI3K, we employed SPR analysis using a Biacore 8 K system. The dissociation constant (K_D_) of RSV for PI3K was 5.73 × 10^−6^ M, confirming a concentration-dependent interaction between RSV and recombinant PI3K (Fig. 8H). Collectively, we suggest that RSV can target and interact with PI3K, further enhancing the stability of PI3K affinity targets.

### *RSV significantly alleviates symptoms of AA by inhibiting ferroptosis *in vivo

**Fig. 9:**
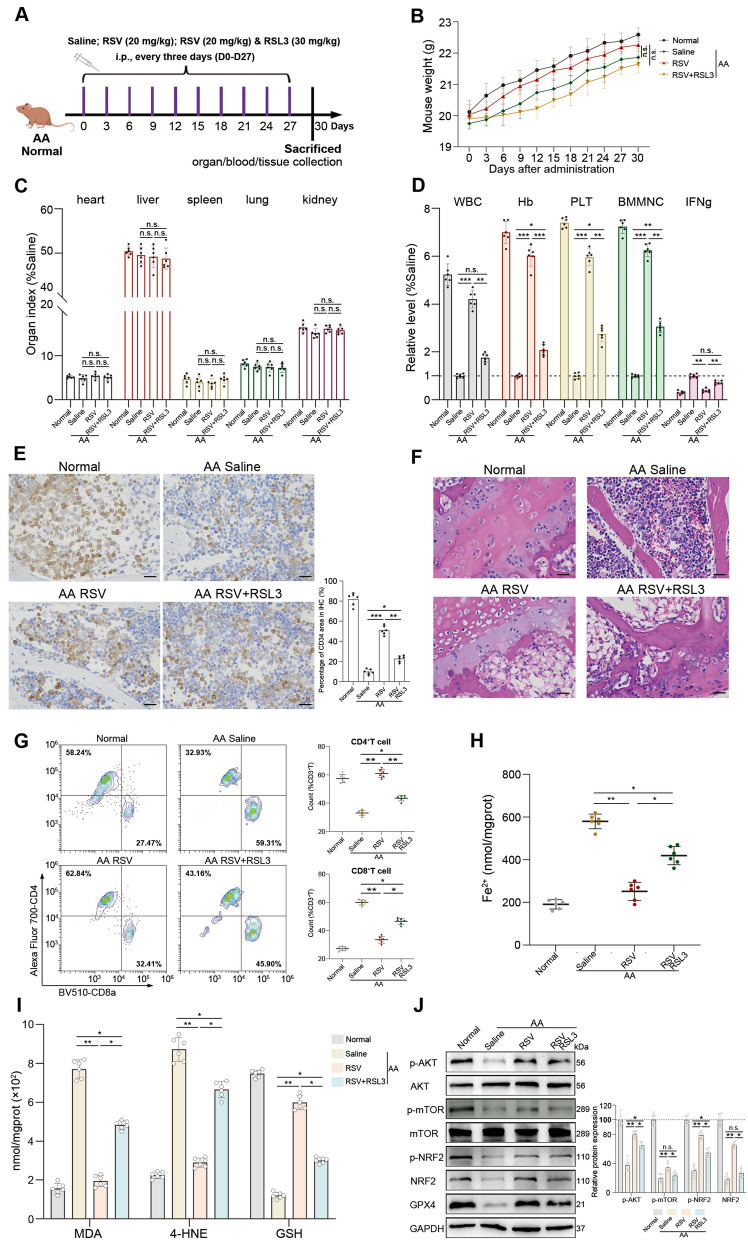
RSV significantly alleviates symptoms of AA by inhibiting ferroptosis in vivo. **A** Illustration of the experimental schedule for the AA mouse model to explore the mechanisms by which RSV relieves symptoms of AA disease. **B** Mouse body weight in each group was measured every 3 days. **C** Organ index of mice was measured in each group, including the heart, liver, spleen, lung, and kidney. **D** ELISA assays were performed to measure the levels of WBC, Hb, PLT, and IFN-gamma in peripheral blood, and BMMNC derived from BM in each mouse. **E** Representative images of IHC of the sternum stained with CD34 in each group, and the quantitative data are presented (scale bar: 25 μm). **F** Representative images of H&E staining of the sternum in each group, and the quantitative data are presented (scale bar: 25 μm). **G** Flow cytometry assays were performed to measure the percentage of CD4^+^ T and CD8^+^ T cells in BM of each mouse, and the quantitative data are presented. **H** Levels of Fe^2+^ in the BM of each mouse were measured using ELISA assays. **I** Levels of MDA, 4-HNE, and GSH in the BM of each mouse were measured using ELISA assays. **J** WB assays were performed to detect the protein levels of p-AKT, AKT, p-mTOR, mTOR, p-NRF2, NRF2 and GPX4 in the BM of each mouse. Data are presented as mean ± SEM; n = 6. Statistical significance was calculated using the “RSV-treated or RSV + RSL3-treated group” against the “Saline group” or the “RSV + RSL3-treated group” against the “RSV-treated group”

To evaluate the RSV-inhibited ferroptosis in AA in vivo, a mouse model of AA was developed and validated for the therapeutic efficacy of RSV. Saline, RSV (20 mg/kg), and RSV (20 mg/kg) + RSL3 (30 mg/kg) were intraperitoneally administered to AA mice or normal mice every 3 days (Fig. 9A). Following ten treatment sessions, the mice were euthanized, and their organs and tissues were excised and weighed. As illustrated in Fig. 9B, RSV has no significant changes in the body weight of AA mice treated with associated drugs compared with the normal mice. The organ index assays demonstrate that the doses of RSV and RSL3 employed in our experiments exhibited no significant biological toxicities on the heart, liver, spleen, lung, or kidney, indicating that the concentrations of drugs utilized in this research have no toxic effect in vivo (Fig. 9C). Additionally, in the group of AA mice undergoing RSV therapy, WBC, Hb, PLT, and BMMNC levels were significantly higher than those of control groups (Saline), which were significantly reversed by RSL3 (Fig. 9D). Notably, in RSV-treated mice, AA symptoms were relieved, accompanied by a significant decrease in IFN-gamma levels.

Subsequently, IHC staining experiments on the sternum revealed that RSV significantly increased CD34 expression, whereas this expression was reduced after RSL3 treatment, resulting in the conclusion that RSV could significantly enhance the activity of hematopoietic cells by inhibiting ferroptosis (Fig. 9E). H&E staining revealed a significant enhancement in sternal histomorphology after RSV treatment (Fig. 9F). To further characterize the therapeutic effect of RSV, we performed flow cytometry experiments to quantify the proportion of CD8^+^ T and CD4^+^ T cells in mouse peripheral blood. Our data demonstrated that the number of CD8^+^ T cells is significantly greater than that of CD4^+^ T cells in AA mice, whereas with RSV treatment, the ratio of CD8^+^ T to CD4^+^ T cells was significantly reversed, reaching a level comparable to that of the normal group (Fig. 9G; Additional file 1: Fig. S5). Further results revealed that RSV decreased Fe^2+^, MDA, and 4-HNE levels and increased GSH production compared to Saline-treated AA mice (Fig. 9H-I). Collectively, these findings suggest that RSV has developed into a novel drug for AA treatment by inhibiting the ferroptosis pathways. Western blotting assays revealed that p-AKT, p-mTOR, p-NRF2, and GPX4 expression were significantly higher in RSV-treated AA mice, whereas these targets in RSV- and RSL3-cotreated AA mice exhibited no significant change compared to Saline-treated AA mice (Fig. 9J). Collectively, our results revealed that RSV inhibits ferroptosis in AA in vivo by inducing the PI3K/AKT/mTOR/NRF2/GPX4 axis, resulting in the suppression of AA development.

## Discussion

AA is a systemic and potentially life-threatening disease associated with an immune-mediated BM failure disorder, often leading to myelodysplasia or myelofibrosis. Clinically, most patients with AA exhibit pronounced symptoms of pancytopenia, including decreases in PLT, WBC, and red blood cells [3]. In general, AA is caused by T-cell-mediated destruction of hematopoietic progenitor cells, leading to reduced activity and loss of function of hematopoietic stem cells, resulting in a severe deficiency of hematopoietic function [7]. Based on AA pathogenesis, it is often clinically manifested as congenital AA, including conditions such as Fanconi anemia, dyskeratosis congenita, Shwachman–Diamond syndrome, Diamond–Blackfan anemia, and congenital amegakaryocytic thrombocytopenia, and acquired AA is characterized by some external factors-induced BM failure, including drugs, radiation damage, and viral infections [2]. Additionally, AA includes inflammatory AA and iron-deficiency AA. The inflammatory AA, termed “anemia of chronic disease,” mediated by immune cell activation and the increased levels of pro-inflammatory cytokines, is characterized by dysregulation of iron metabolism, relative insufficiency of EPO secretion, and impaired BM erythropoiesis [32]. Iron deficiency anemia has emerged as a recent research hotspot, primarily caused by insufficient iron intake or depletion of iron stores, leading to iron deficiency in red blood cells, severely affecting the function of red blood cells, and causing anemia [33].

Ferroptosis is an iron-dependent type of regulated cell death, characterized by LIP of membrane phospholipids, reliant on intracellular reactive oxygen species, and mechanistically and morphologically distinct compared to other cell death modalities [19]. A growing body of research indicates that ferroptosis in cells is frequently accompanied by changes in mitochondrial morphology, including mitochondrial shrinkage, increased MMP, and diminished mitochondrial cristae. Recent research indicates that the disruption of iron homeostasis occurs in various inflammatory diseases, including rheumatoid arthritis, psoriasis, diabetes, and inflammatory bowel disease [34]. There is increasing evidence that iron metabolism significantly contributes to AA development and exacerbates BM stem cell failure. Yu et al. reported that targeting the intestinal Hif2α-Fpn axis, crucial for inhibiting intestinal iron absorption, may present a viable therapeutic approach for treating various types of anemia, including iron-refractory anemia, inflammatory anemia, and chemotherapy-induced anemia [35]. A recent study performed by Xiao et al. demonstrated that butyrate upregulates SLC40A1 expression by reducing the acetylation of transcription factors at the Slc40a1 promoter, thereby facilitating iron export in macrophages [36]. This study reveals the crucial function of butyrate in mitigating colitis-induced anemia and diminishing TNF-α production in macrophages, thus preventing the pathogenic circuit between anemia and inflammation. Based on the above two studies, although it was demonstrated that iron ion homeostasis was abnormal for AA disease, which was closely related to the ferroptosis process, the researchers did not focus on alterations in ferroptosis indicators during AA. Additionally, Lu et al. revealed that the role of the a GATA1/SREBP2/NFE2 axis provides a feedback loop to regulate the cholesterol homeostasis during erythroblast differentiation, providing new insights into the functions of lipid metabolism involved in ferroptosis in erythropoiesis [37]. Therefore, we can confidently propose a substantial hypothesis that there is a strong positive correlation between ferroptosis and AA, which requires additional investigation. In support of this hypothesis, using in vitro and in vivo experiments, we have demonstrated that the ferroptosis pathway is essential in AA progression by inhibiting the PI3K/AKT/mTOR/NRF2/GPX4 axis.

Clinically, we observed that the accumulation of IFN-gamma, secreted by activated CTLs, can exacerbate the symptoms in patients with AA. Notably, several studies have demonstrated that IFN-gamma significantly facilitates ferroptosis in numerous diseases. For example, Liao et al. reported that T cell-derived IFN-gamma can stimulate ACSL4 expression, inducing immunogenic tumor ferroptosis by facilitating the metabolic pathway of long-chain polyunsaturated fatty acids, presenting as a novel mode of action for CD8^+^ T cell-mediated cytotoxicity [38]. Cao et al. have demonstrated that IFN-gamma activated STAT1 phosphorylation and downregulated SLC3A2 and GPX4 expression in salivary gland epithelial cells in Sjogren's syndrome, attenuating the ability to combate intracellular oxidative stress and subsequently inducing ferroptosis [39]. The above studies indicate that the pro-inflammatory cytokine IFN-gamma is considered crucial in developing and treating various diseases, including AA. We provided a novel perspective that abnormally activated cytokine IFN-gamma in AA can exacerbate the hematopoietic cell failure by inducing ferroptosis. These findings enhance AA pathogenesis and hold significant scientific implications for understanding the important role of ferroptosis in AA. However, in our study, the cell line used for the in vitro experiments, 32D, has certain limitations, mainly that it cannot fully replicate the vulnerability of human multipotent HSPCs in AA. Therefore, in future research, we will further test the therapeutic effects of RSV on primary human CD34^+^ cells or patient-derived HSPCs to strengthen the translational aspect of our findings and help advance the clinical use of RSV for treating AA.

Previous reports revealed that RSV exhibited potential activity in regulating cellular ferroptosis, which is implicated in several diseases. Notably, the functional activity was defined by two aspects, including tumor control by inducing ferroptosis and alleviating autoimmune diseases by inhibiting ferroptosis. Our previous study offers novel insights into the biological role of RSV in treating triple-negative breast cancer (TNBC), wherein GPX4 protein was diminished through enhanced NEDD4L-mediated ubiquitination, proposing a new treatment program for the clinical treatment of TNBC [40]. However, multiple studies have confirmed that RSV can effectively inhibit ferroptosis to alleviate the symptoms of autoimmune diseases. For instance, Wang et al. have confirmed that RSV-inhibited ferroptosis can ameliorate intestinal ischemia–reperfusion injury by activating the SIRT3/FoxO3a signaling pathway, hence decreasing ROS production and enhancing the activity of GSH/GPX4 pathway [41]. Li et al. have demonstrated that RSV effectively mitigates depression-like behaviors by inhibiting ferroptosis through promoting the AKT/NRF2 signaling pathway [42]. Ni et al. have elucidated that RSV inhibits ferroptosis via activating the NRF2/GPX4 signaling pathway in mice with spinal cord injuries, facilitating the recovery of neurological and motor function [43]. A recent study conducted by Zhang et al. demonstrated that RSV activation of the Sirt1/p53 pathway inhibits ferroptosis by reducing the depletion of SLC7A11, thereby enhancing cardiac function by inhibiting ferroptosis [44]. Consequently, RSV has an important function in the regulation of ferroptosis based on previous research. Additionally, a preliminary study showed that RSV has the potential to protect hematopoietic stem cells from radiation via activation of Sirt1, suggesting that it may be an effective therapeutic agent for ameliorating TBI-induced long-term BM injury [45]. In this study, for the first time, our data functionally demonstrate that RSV treatment stabilizes NRF2 protein, resulting in a significant increase in GPX4 expression and subsequent amelioration of AA symptoms by inhibiting ferroptosis.

## Conclusion

**Fig. 10:**
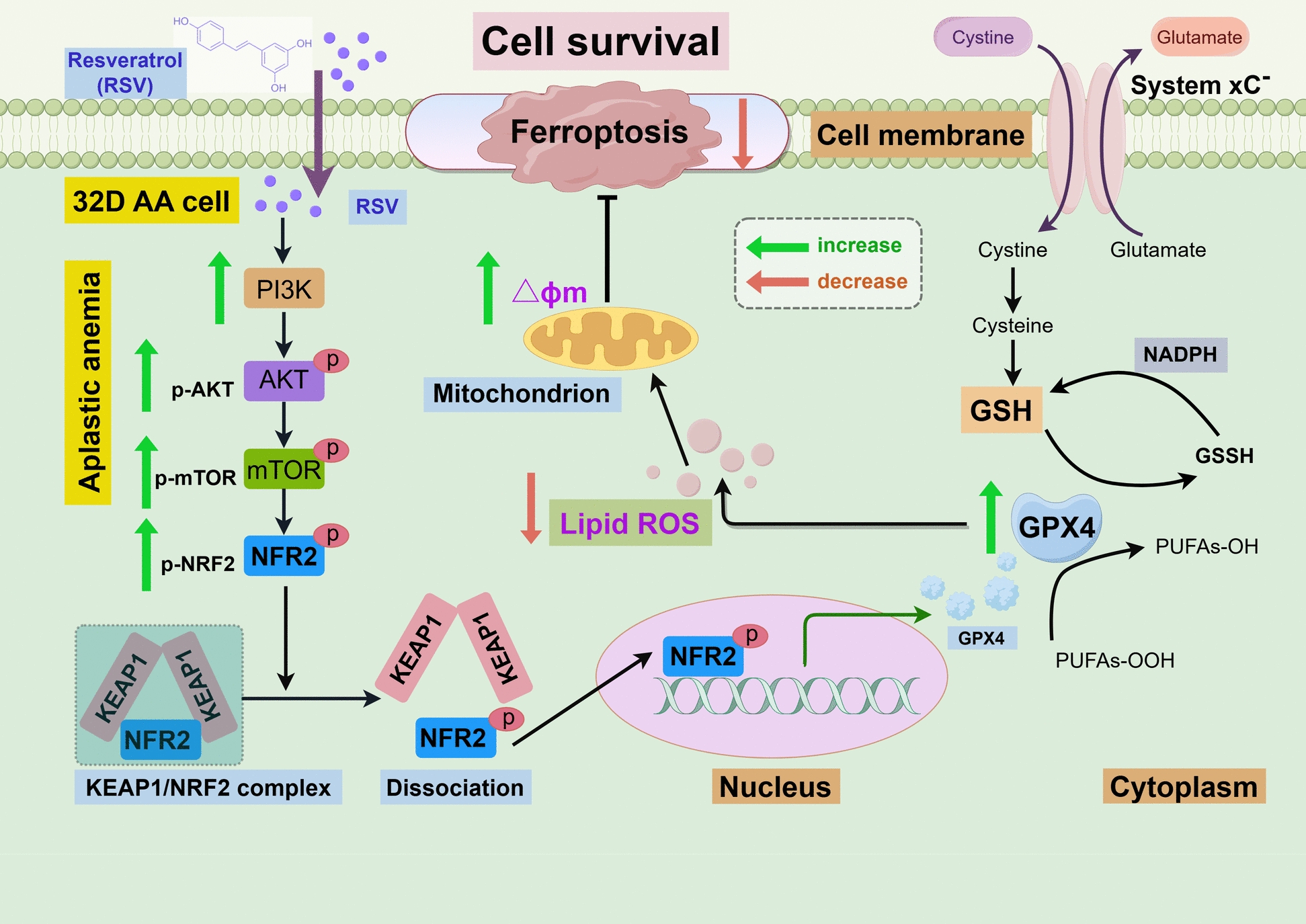
Schematic representation of the mechanisms by which RSV inhibits ferroptosis via the PI3K/AKT/mTOR/NRF2/GPX4 axis to mitigate IFN-gamma-mediated AA. The illustration demonstrates that RSV-mediated the activation of PI3K facilitates the phosphorylation of AKT (p-AKT) and mTOR (p-mTOR), promoting the levels of phosphorylated NRF2 (p-NRF2) in AA. The enhanced level of p-NRF2 enhances the dissociation of KEAP1 and NRF2 complex and subsequently increases GPX4 expression, contributing to the beneficial effect of RSV for the inhibition of AA progression in a ferroptosis-dependent manner

In summary, RSV can significantly inhibit ferroptosis in AA or IFN-gamma-treated 32D cells via targeting the PI3K/AKT/mTOR signaling pathway, hence promoting NRF2 phosphorylation and subsequent GPX4 transcription, alleviating AA symptoms (Fig. 10). One of the limitations of our study is that Nrf2 or Gpx4 knockout AA mice may require additional testing to investigate the precise mechanism of RSV. In addition, the therapeutic effect of RSV in clinical AA warrants further investigation. The findings of our study offer substantial evidence that targeting the PI3K/AKT/mTOR/NRF2/GPX4 axis by RSV serves as a potential therapeutic strategy for treating ferroptosis-related AA.

## Supplementary Information

Supplementary Material 1.  Table S1. Primers in this research. Fig. S1. The quantification of mean fluorescence intensity for ROS and LIP in 32D cells in the absence or presence of IFN-gamma for 24 h. Fig. S2. The quantification of damaged mitochondria in 32D cells treated with indicated concentrations of IFN-gamma for 24 h. Fig. S3. The quantification of damaged mitochondria in 32D AA cells treated with indicated concentrations of RSV for 24 h. Fig. S4. The quantification of mean fluorescence intensity for ROS and LIP in 32D cells transfected with siNC or siGPX4 plasmids in the absence or presence of RSV for 24 h. Fig. S5. The ratio of CD4^+^ T to CD8^+^ T cells in BM of each mouse.

## Data Availability

All data generated or analyzed during this study are included in this published article (and its supplementary information files).

## Abbreviations

AA: Aplastic anemia
HSCT: Hematopoietic stem cell transplantation
IST: Immunosuppressive treatment
ROS: Reactive oxygen species
LIP: Lipid peroxidation
PND: Panaxadiol saponin
NRF2: Nuclear factor erythroid 2-related factor 2
HO-1: Heme oxygenase-1
PI3K: Phosphatidylinositol 3-kinase
AKT: Protein kinase B
mTOR: Mammalian target of rapamycin
CTLs: Cytotoxic T lymphocytes
RSV: Resveratrol
ARE: Antioxidant response element
GPX4: Glutathione peroxidase 4
SIRT1: Sirtuin 1
KEAP1: Kelch-like ECH-associated protein 1
GSH: Glutathione
MMP: Mitochondrial membrane potential
TEM: Transmission electron microscopy
ELISA: Enzyme-linked immunosorbent assay
MDA: Malondialdehyde
4-HNE: 4-Hydroxynonenal
GSH: Glutathione
WB: Western blotting
CHX: Cycloheximide
Baf A1: Bafilomycin A1
CQ: Chloroquine
CETSA: Cellular thermal shift assay
PIK3CA: Phosphatidylinositol 4,5-bisphosphate 3-kinase catalytic subunit alpha isoform
DARTS: Drug affinity responsive target stability
SPR: Surface plasmon resonance
HD: Healthy donor
BMMNCs: Bone marrow mononuclear cells
TBI: Total body irradiation
WBC: White blood cell
Hb: Hemoglobin
PLT: Platelet
BM: Bone marrow
COX2: Cyclooxygenase 2
TNBC: Triple-negative breast cancer
