# Supplementary material for: Resveratrol-activated PI3K/AKT/mTOR signaling pathway attenuates ferroptosis by promoting NRF2/GPX4 metabolic pathway to mitigate IFN-gamma-mediated aplastic anemia
Source: Chin Med. 2026 Jul 8;21:185. doi: 10.1186/s13020-026-01462-5 (PMC13343987; doi:10.1186/s13020-026-01462-5)
Supplement: Supplementary file 1 — Supplementary Material 1. Table S1. Primers in this research. Fig. S1. The quantification of mean fluorescence intensity for ROS and LIP in 32D cells in the absence or presence of IFN-gamma for 24 h. Fig. S2. The quantification of damaged mitochondria in 32D cells treated with indicated concentrations of IFN-gamma for 24 h. Fig. S3. The quantification of damaged mitochondria in 32D AA cells treated with indicated concentrations of RSV for 24 h. Fig. S4. The quantification of mean fluorescence intensity for ROS and LIP in 32D cells transfected with siNC or siGPX4 plasmids in the absence or presence of RSV for 24 h. Fig. S5. The ratio of CD4+ T to CD8+ T cells in BM of each mouse. [file 13020_2026_1462_MOESM1_ESM.docx]

**Additional file 1**

**Resveratrol-activated PI3K/AKT/mTOR signaling pathway attenuates ferroptosis by promoting NRF2/GPX4 metabolic pathway to mitigate IFN-gamma-mediated aplastic anemia**

Erhao Zhang^1,2,*^, Jianan Cui^1,2^, Xinran Zhu^1^, Juan Chen^1^, Liqin Xu^3^, Xiaomin Li^1,2^, Yufei Zhang^1,2^, Hui Jia^1^, Xiaorong Zhou^1,2^, Zenghua Lin^1,*^, Han Wang^4,*^, Hong Liu^1,*^

^1^Department of Hematology, Affiliated Hospital of Nantong University, Medical School of Nantong University, Nantong 226001, P.R. China

^2^Department of Immunology, Medical School of Nantong University, Nantong 226001, P.R. China

^3^Department of Pulmonary and Critical Care Medicine, Affiliated Hospital of Nantong University, Medical School of Nantong University, Nantong 226001, P.R. China

^4^Department of General Surgery, Affiliated Hospital of Nantong University, Medical School of Nantong University, Nantong 226001, P.R. China

**Correspondence:** Erhao Zhang, E-mail: zhangerhao@ntu.edu.cn; Zenghua Lin, E-mail: linzenghua@126.com; Han Wang, wwhh1991@aliyun.com; Hong Liu, E-mail: hongliu63@126.com

**Table S1.** Primers in this research.

| **Primer name** | **Sequence (5’→3’)** | **Application** |
| --- | --- | --- |
| M-CASP3-RTF | gagatggcttgccagaagat | qPCR |
| M-CASP3-RTR | taacgcgagtgagaatgtgc | qPCR |
| M-CASP8-RTF | agtgagcagatcagaattgagg | qPCR |
| M-CASP8-RTR | gggcacagactcttttcagg | qPCR |
| M-LC3-RTF | gcctgtcctggataagacca | qPCR |
| M-LC3-RTR | ccgtcttcatccttctcct | qPCR |
| M-P62-RTF | caccaatgccatctcaagtg | qPCR |
| M-P62-RTR | gagctacccacagagccaag | qPCR |
| M-RIPK1-RTF | gtcggacgtgtacagctttg | qPCR |
| M-RIPK1-RTR | gaggctgatgatctcccttg | qPCR |
| M-RIPK3-RTF | ctggtgagccgtgaagaact | qPCR |
| M-RIPK3-RTR | gacacgaagtcccactggag | qPCR |
| M-MLKL-RTF | ccccctgagttctccattgt | qPCR |
| M-MLKL-RTR | gaggaaactggagctgctga | qPCR |
| M-GSDMD-RTF | tgtctggtgcttgactctgg | qPCR |
| M-GSDMD-RTR | caactccagctgctttgaca | qPCR |
| M-CASP1-RTF | tggcattaagaaggcccata | qPCR |
| M-CASP1-RTR | tcctccaagtcacaagacca | qPCR |
| M-NLRP3-RTF | agacaactgcagcctcacct | qPCR |
| M-NLRP3-RTR | tcttcctggagcgcttctaa | qPCR |
| M-SLC7A11-RTF | cccagatatgcatcgtcctt | qPCR |
| M-SLC7A11-RTR | cgtctgaaccacttgggttt | qPCR |
| M-GPX4-RTF | tctcagccaaggacatcgac | qPCR |
| M-GPX4-RTR | ggccaggattcgtaaaccac | qPCR |
| M-NRF2-RTF | gcgacagaaggactatgagc | qPCR |
| M-NRF2-RTR | tccactggtgtctgtctgga | qPCR |
| M-KEAP1-RTF | acgggactaaccggcttaac | qPCR |
| M-KEAP1-RTR | gtagcgctccacactgttca | qPCR |
| M-PTGS2-RTF | caggtgatgctgacagagga | qPCR |
| M-PTGS2-RTR | gagagtgaggacccactgga | qPCR |
| M-GAPDH-RTF | gtgaaggtcggtgtgaacg | qPCR |
| M-GAPDH-RTR | tgccgtgagtggagtcatac | qPCR |
| M-siGPX4-1 | aguuuacgucaguuuugccuc | siRNA |
| M-siGPX4-2 | ucaaacuugacguuguagccg | siRNA |
| M-siGPX4-3 | agaaacuugguaaaguuccau | siRNA |
| M-siNRF2-1 | acuuacuccaagaucuauguc | siRNA |
| M-siNRF2-2 | uuaagacacuguaauucggga | siRNA |
| M-siNRF2-3 | ugaaagaauccucaaaaccau | siRNA |
| M-sgNRF2-1 | ttactcatcgatctcctcgc | sgRNA |
| M-sgNRF2-2 | agccttcaatagtcccgtcc | sgRNA |
| M-sgNRF2-3 | atgtgctgggccggctgaat | sgRNA |
| M-GPX4-F | tgtgattgtgggctttaagacc | CUT&RUN; Dual Luciference |
| M-GPX4-R | tttactgctgtgaacagacacc | CUT&RUN; Dual Luciference |

**Fig. S1.** The quantification of mean fluorescence intensity for ROS and LIP in 32D cells in the absence or presence of IFN-gamma for 24 h. Data are presented as mean ± SEM; n = 3. Statistical significance was calculated using the “IFN-gamma-treated group” against the “DMSO group”.

**Fig. S2.** The quantification of damaged mitochondria in 32D cells treated with indicated concentrations of IFN-gamma for 24 h. Data are presented as mean ± SEM; n = 3. Statistical significance was calculated using the “IFN-gamma-treated group” against the “DMSO group”.

**Fig. S3.** The quantification of damaged mitochondria in 32D AA cells treated with indicated concentrations of RSV for 24 h. Data are presented as mean ± SEM; n = 3. Statistical significance was calculated using the “RSV-treated group” against the “DMSO group”.

**Fig. S4.** The quantification of mean fluorescence intensity for ROS and LIP in 32D cells transfected with siNC or siGPX4 plasmids in the absence or presence of RSV for 24 h. Data are presented as mean ± SEM; n = 3. Statistical significance was calculated using the “experimental group” against the “si-NC/DMSO group”.

**Fig. S5.** The ratio of CD4^+^T to CD8^+^T cells in BM of each mouse. Data are presented as mean ± SEM; n = 6. Statistical significance was calculated using the “RSV-treated or RSV+RSL3-treated group” against the “Saline group”.
